# Proteomics and Phosphoproteomics Revealed Dysregulated Kinases and Potential Therapy for Liver Fibrosis

**DOI:** 10.1016/j.mcpro.2025.100991

**Published:** 2025-05-12

**Authors:** Xinyu Cheng, Li Kang, Jinfang Liu, Qingye Wang, Zhenpeng Zhang, Li Zhang, Yuping Xie, Lei Chang, Daobing Zeng, Lantian Tian, Lingqiang Zhang, Ping Xu, Yanchang Li

**Affiliations:** 1 Anhui Medical University School of Basic Medicine, Anhui, PR China; 2 State Key Laboratory of Medical Proteomics, Beijing Proteome Research Center, National Center for Protein Sciences (Beijing), Beijing Institute of Lifeomics, Beijing, China; 3 School of Public Health, China Medical University, Shenyang, China; 4 TaiKang Medical School (School of Basic Medical Sciences), Key Laboratory of Combinatorial Biosynthesis and Drug Discovery of Ministry of Education, School of Pharmaceutical Sciences, Wuhan University, Wuhan, PR China; 5 General Surgery Department, Beijing Youan Hospital, Capital Medical University, Beijing, China; 6 Department of Hepatobiliary and Pancreatic Surgery, the Affiliated Hospital of Qingdao University, Qingdao, Shandong, PR China; 7 College of Life Sciences, Hebei University, Baoding, China

**Keywords:** proteomics, phosphoproteomics, kinases, liver fibrosis, inhibitors

## Abstract

Liver fibrosis is the initial stage of most liver diseases, and it is also a pathological process involving the liver in the late stages of many metabolic diseases. Therefore, it is important to systematically understand the pathological mechanism of liver fibrosis and seek therapeutic approaches for intervention and treatment of liver fibrosis. Disordered proteins and their post-translational modifications, such as phosphorylation, play vital roles in the occurrence and development of liver fibrosis. However, the regulatory mechanisms that govern this process remain poorly understood. In this study, we analyzed and quantified the liver proteome and phosphoproteome of carbon tetrachloride–induced early liver fibrosis model in mice. Proteomic analysis revealed that the pathways involved in extracellular matrix recombination, collagen formation, metabolism and other related disorders, and protein phosphorylation modification pathways were also significantly enriched. In addition, Western blotting and phosphoproteomics demonstrated that phosphorylation levels were elevated in the context of liver fibrosis. A total of 13,152 phosphosites were identified, with 952 sites increased, whereas only 156 sites decreased. Furthermore, the upregulated phosphorylation sites, which exhibited no change at the proteome level, mainly shared a common [xxxSPxxx] motif. Consequently, the kinase–substrate analysis ascertained the overactive kinases of these upregulated substrates, which ultimately led to the identification of 13 significantly altered kinases within this dataset. These kinases were mainly cataloged into the STE, CMGC, and CAMK kinase families. Among them, STK4 (serine/threonine-protein kinase 4), GSK3α (glycogen synthase kinase 3α), and CDK11B (cyclin-dependent kinase 11B) were subsequently validated though cellular and animal experiments, and the results demonstrated that their inhibitors could effectively reduce the activation of hepatic stellate cells and extracellular matrix production. These kinases may represent potential therapeutic targets for liver fibrosis, and their inhibitors may serve as promising antihepatic fibrosis drugs.

## Highlights

• Proteomic and phosphoproteomic analysis associated the early hepatic fibrosis with disordered phosphorylation.
• Three dysregulated kinases, STK4, GSK3α, and CDK11B, were screened out in mouse liver fibrosis.
• Dysregulated kinase inhibitors alleviated early hepatic fibrosis progression, indicating potential antifibrotic therapy.

## Graphical Abstract

**Figure:**
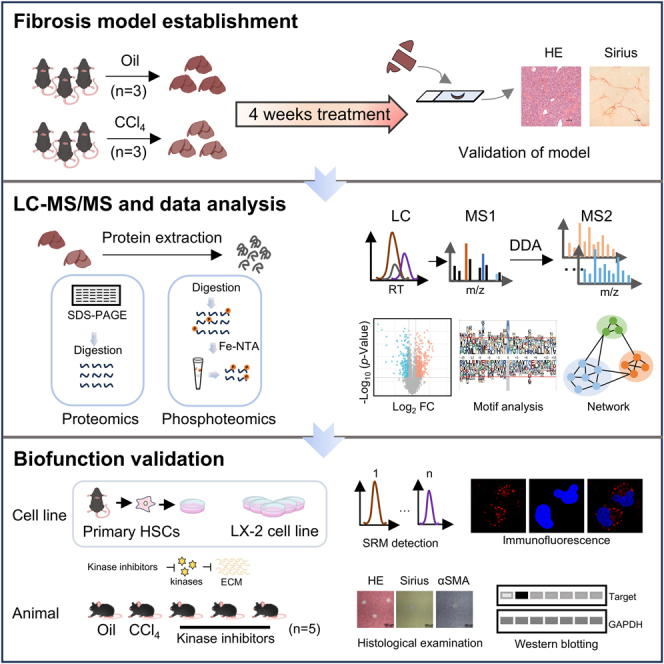

## In Brief

Proteomic and phosphoproteomic analysis on mouse hepatic fibrotic liver induced with CCl_4_ revealed that the liver fibrosis was closely associated with extensive dysregulation of phosphorylation signal. To inhibit the corresponding upstream dysregulated kinases could alleviate the progression of liver fibrosis. This study provides a potential therapeutic approach of targeting specific phosphokinases for treating early stage liver fibrosis.

Liver fibrosis is an abnormal pathological response to chronic liver injury, characterized by accumulation of extracellular matrix (ECM) proteins within the subcutaneous space of Disse, with excessive deposition of collagenous and noncollagenous components, resulting in the area of liver injury being surrounded by new dense ECM scars (1, 2). Various chronic liver insults, including viral (hepatitis B or C), metabolic (nonalcoholic steatohepatitis), autoimmune, and toxic (*e.g.*, alcohol, *N*-nitrosodimethylamine) injuries, can lead to the development of liver fibrosis (3, 4, 5). Persistent fibrotic lesions can lead to progressive loss of liver function and development of cirrhosis or even hepatocellular carcinoma. It can be said that liver fibrosis is a necessary part of the development of the vast majority of end-stage liver diseases. Many studies have shown that early liver fibrosis could be reversible, although its molecular mechanisms are not fully understood (5, 6). Therefore, earlier diagnosis and earlier intervention of liver fibrosis are important to stop or delay the progression of lethal liver diseases (7).

The molecular mechanisms of hepatic fibrogenesis have been extensively revealed by studies in patients with hepatic fibrosis of various etiologies and by experimental models of hepatic fibrosis in rodents. These mechanisms include chronic hepatocellular injury, epithelial or endothelial barrier damage, release of inflammatory cytokines, recruitment of bone marrow–derived inflammatory cells, production of transforming growth factor beta (TGF-β) by macrophages, activation of hepatic stellate cells (HSCs), overproduction of ECM, and formation of fibrous scars (8). Our recent study has identified cathepsin S upregulation as a central node of ECM remodeling in human fibrotic livers by proteomic screening and demonstrated cathepsin S as a potential target for the diagnosis and treatment of liver fibrosis by exploring its mechanism (9). Currently, despite a series of research advances in the pathophysiological mechanisms of liver fibrosis, there are still gaps in identifying antihepatic fibrosis targets and translating them into effective therapeutic approaches, and the search for molecular targets that can attenuate or reverse liver fibrosis is still of great scientific and clinical importance.

Protein phosphorylation modifications, coregulated by protein kinases and phosphatases, are one of the most important post-translational modifications as molecular switches for intracellular signaling that play a key role in almost all biological processes (10, 11). Numerous studies have shown that phosphorylation plays an important role in the progression of liver fibrosis (12, 13). In addition, upstream kinases that regulate protein phosphorylation modifications, which may serve as potential antifibrotic targets, have received widespread attention from researchers (14, 15, 16). For example, Martin *et al.* (17) have shown that P21-activated kinase (PAK) and mechanosensitive factor (YAP-1) are central mediators of profibrotic integrin beta-1 signaling, and that inhibition of PAK activity markedly inactivates the profibrotic myofibroblast phenotype and limits scarring induced by various liver insults. As another example, STAT3 is involved in the development of liver fibrosis, and a study by Su *et al.* (18) found that sorafenib and its derivative SC-1 activated Src-homology protein tyrosine phosphatase (SHP-1) and inhibited STAT3, which may represent a new strategy for antifibrotic drug discovery. In contrast, in another study, it was shown that ruxolitinib targeted and inhibited the STAT3 upstream kinase Janus kinase 1/2, which could inhibit the progression of carbon tetrachloride (CCl_4_)–induced liver fibrosis in mice (19, 20). Although studies have shown that phosphorylation plays a vital role in liver fibrosis, its regulatory mechanisms have not been fully profiled and understood. Although no drugs have been approved for the treatment of liver fibrosis, a large number of studies have suggested that kinases may be important drug targets for the treatment of liver fibrosis.

Mass spectrometry (MS)–based proteomics or phosphoproteomics has become an increasingly powerful tool for identifying differentially expressed proteins and phosphorylation modifications in complex protein mixtures, which can significantly shorten the discovery cycle of potential therapeutic targets (21, 22). To systematically understand the changes in liver proteins during the process of early liver fibrosis, this study successfully established a mouse model of early liver fibrosis using CCl_4_ treatment. Through proteomic and phosphoproteomic analysis, we found that sites with significantly upregulated phosphorylation levels shared a common [xxxSPxxx] motif, and ultimately 13 significantly altered kinases were identified in this dataset. Our study further validated the aberrant activation of STK4 (serine/threonine-protein kinase 4), GSK3α (glycogen synthase kinase 3α), and CDK11B (cyclin-dependent kinase 11B) in liver fibrosis mice and found that their small-molecule inhibitors have antihepatic fibrosis effects.

## Experimental Procedures

### CCl_4_-Induced and Kinase Inhibitors' Administration Mouse Model

C57BL/6 mice (6–8 weeks old) were obtained from SPF Biotechnology. The mice were housed in an SPF barrier facility under a 12-h light–dark cycle at a temperature of 21 ± 1 °C and humidity of 45 ± 10%, with free access to water and standard chow. To induce liver fibrosis, CCl_4_ was administered intraperitoneally every 2 days for 4 weeks (200 μl per mouse, diluted 1:7 in olive oil), whereas control mice received the vehicle alone (9).

To inhibit kinase activity, mice were treated with CCl_4_ as described previously for 2 weeks, and inhibitors (SBP3264 at 10 mg/kg, laduviglusib at 6 mg/kg or 18 mg/kg, OTS964 at 5 mg/kg or 15 mg/kg) were administered intraperitoneally every other day during the hepatic fibrosis process. This project was approved by the Institute Animal Care and Use Committee of Beijing Proteome Research Center (ethics review number: IACUC-20200303-08MO). The livers were perfused to minimize potential blood contamination. The mouse livers were then dissected, rinsed twice with cold PBS, and immediately frozen in liquid nitrogen. Samples were stored at −80 °C until protein extraction. Serum and liver samples were collected for analysis. Serum alanine transaminase and aspartate transaminase activities were measured using an automated biochemical analyzer (HITACHI).

### Liver Tissue From Cirrhotic Patients

Liver tissues from two patients with liver cirrhosis and two normal liver tissues were obtained *via* biopsy from Beijing You’An Hospital. The study was approved by the Institutional Review Board of Beijing You’An Hospital (ethics review number: [2018] 006), with informed consent obtained from patients for sample collection. All human studies conducted strictly adhere to the principles of the Declaration of Helsinki. To protect the privacy of participants, all were fully informed of the research purpose, procedures, potential risks, and their rights prior to the collection of biological samples, and signed a written informed consent. All samples were then anonymized, removing any information that could directly or indirectly identify the individual, such as name, address, and social security number. Subsequently, all liver samples were examined by senior pathologists at the hospital. Following formalin fixation, tissue samples underwent dehydration and were sectioned for immunohistochemical verification.

### Histology Analysis

To validate the hepatic fibrosis model, paraffin-embedded liver samples were sectioned and stained with H&E and Sirius red according to the manufacturer’s protocols. To validate the kinase expression level in patients with cirrhosis, tissue sections were evaluated by immunohistochemistry using anti-STK4 (Beyotime; 1:1000 dilution, #AG3339) and anti-GSK3α (Beyotime; 1:1000 dilution, #AG2065), respectively. Sections without antibody were used as negative controls. For validation of the kinase inhibitor model, tissue sections were evaluated with anti-αSMA (Cell Signaling Technology; 1:600 dilution). The positive area was quantified using the ImageJ 1.48v software (National Institutes of Health).

### Primary HSC Isolation and Stimulation *In Vitro*

Primary mouse HSCs were isolated from wild type C57BL/6 mice, as previously described with the minor modification (9). Liver tissues were first removed and minced with ophthalmic scissors in Dulbecco’s modified Eagle’s medium and then enzymatically digested using collagenase IV and DNase I. Cell suspensions were filtered through a 100 μm nylon mesh, followed by centrifugation at 50*g* for 3 min at 4 °C. The resultant supernatant was then subjected to further centrifugation at 320*g* for 8 min to obtain nonparenchymal liver cells. The HSCs were isolated using a 15% OptiPrep solution. Briefly, the nonparenchymal liver cells were resuspended in 10 ml of 15% OptiPrep and overlaid on the top by gentle addition of 2 ml of Dulbecco’s modified Eagle’s medium. Subsequent to centrifugation at 1400*g* for 20 min, the HSCs were aspirated from the interphase layer and cultivated in RPMI1640 containing 10% fetal bovine serum, 1% penicillin, and 1% streptomycin at 37 °C in a humidified atmosphere of 5% CO_2_.

Following an 8-h culture period of isolated primary HSCs, the addition of 10 ng/ml of the recombinant TGF-β1 protein (Sino Biological; #10804-HNAC) occurred, either as a standalone treatment or in conjunction with 200 nM of the STK4 inhibitor SBP-3264 (MCE; #HY-132969), 100 nM GSK3α inhibitor laduviglusib (MCE; #HY-10182A), or 100 nM CDK11B inhibitor OTS964 (MCE; #HY-12467), respectively. Following a 24-h culture period, cells were harvested for subsequent Western blotting (WB) analysis.

### Cell Culture and Treatment

Human HSC line LX-2 were maintained in RPMI1640 containing 10% fetal bovine serum, 1% penicillin, and 1% streptomycin at 37 °C in a humidified atmosphere of 5% CO_2_. In order to better activate the cells toward fibrosis, cells were starved in serum-free medium for 12 h. Subsequently, 10 ng/ml TGF-β1 was added separately or incubated with 200 nM STK4 inhibitor SBP-3264, 100 nM GSK3α inhibitor laduviglusib, or 100 nM CDK11B inhibitor OTS964, respectively. Cells were harvested after 48 h of culture for subsequent WB analysis.

### Protein Extraction

Mouse liver samples were lysed using lysis buffer containing 9 M urea, 30 mM NaCl, 10 mM Tris–HCl (pH 8.0), 5 mM Na_4_P_2_O_7_, 100 mM NaH_2_PO_4_, 1 mM NaF, 1 mM Na_3_VO_4_, 1 mM sodium glycerophosphate, 5 mM iodoacetamide, 1% phosphatase inhibitor cocktails 2 and 3, and a protease inhibitor cocktail. The cryogenic freeze grinder (Shanghai Jingxin) was utilized to grind the sample 10 times with steel balls, vortexing for 30 s, stopping for 30 s, and repeating 10 cycles. After grinding, the steel balls were removed, and low-temperature ultrasound was performed for 10 min using a noncontact ultrasonic machine (Ningbo Scientz Biotechnology Co, LTD) (worked for 4 s, stopped for 4 s with a total time of 10 min at 80% intensity). The protein concentration of the supernatant was estimated using Pierce BCA Protein Assay Kits (Thermo Fisher Scientific).

For the LX-2 cell, precooled lysis solution (8 M urea, 150 mM NaCl, 50 mM Tris–HCl [pH 8.0], 0.2% NP-40, 10% glycerol, 1 mM PMSF, 10 mM Tris(2-carboxyethyl)phosphine hydrochloride, and 40 mM chloroacetamide) was added to the cell pellets. Subsequently, the cells were lysed at low temperature using a noncontact ultrasonic crusher (operating for 10 s, paused for 20 s, with a total working time of 15 min and a power of 60%). After ultrasonic cracking, lysates were centrifuged at 21,000*g* at 4 °C for 5 min to eliminate insoluble debris, and the supernatant was utilized for the subsequent WB.

### SDS-PAGE and WB Analysis

The protein concentration of the supernatant was estimated using a bicinchoninic acid protein assay. SDS sample-loading buffer (250 mM Tris–HCl [pH 6.8], 20% [v/v] glycerol, 0.5% [w/v] SDS, and 0.02% [w/v] bromophenol blue) was added to the lysates before separated by SDS-PAGE.

Proteins were separated on a 10% SDS-PAGE and transferred onto a nitrocellulose membrane using the Bio-Rad Trans-Blot SD Semi-Dry Transfer System for 55 min at 15 V. The membranes were blocked in 5% bovine serum albumin (BSA) in Tris-buffered saline with Tween-20 (TBST) (20 mM Tris, pH 7.4, 150 mM NaCl, containing 0.02% Tween-20) for 2 h. For analysis of global phosphorylation, we used the phosphoserine/threonine/tyrosine polyclonal antibodies (Thermo Fisher Scientific; 1:1000 dilution, #61-8300). For analysis of the subsequent biological verification, WB analysis was performed with anti-COL1A1 (Cell Signaling; 1:1000 dilution, #72026), anti-αSMA (Cell Signaling; 1:1000 dilution, #19245), anti-STK4 (Beyotime; 1:1000 dilution, #AG3339), anti-GSK3α (Beyotime; 1:1000 dilution, #AG2065), and anti-GAPDH (ABclonal; #AC002). The membranes were washed three times with TBST for 10 min each to remove excess primary antibody. After washing, the membranes were incubated with a peroxidase-conjugated secondary antibody diluted at 1:10,000 in 5% BSA in TBST for 1 h at room temperature. Excess secondary antibody was removed by washing the membranes three times for 10 min each in TBST. The membranes were exposed to the Super Signal West Pico chemiluminescent substrate (Thermo Fisher Scientific) for 1 min at room temperature and visualized using a Tanon 5200 chemiluminescence imaging analysis system (Tanon). Detection and quantification of the band intensities were conducted using ImageJ 1.48v software. Nitrocellulose membranes were stripped in antibody removal solution (CWBIO) for 20 min and washed three times in TBST before restarting the blocking and immunoblotting procedure.

### Quantitative RT–PCR Analysis

Total RNA was extracted from mouse liver using TRIzol reagent (Invitrogen). To eliminate genomic DNA contamination, additional DNase treatment was performed with RNase-free DNase I (Promega). About 1.2 μg of RNA was reverse transcribed into complementary DNA in a 20 μl reaction mixture using the Revertra Ace quantitative PCR (qPCR) RT Kit (TOYOBO). mRNA level of each gene was determined by quantitative real-time PCR using SYBR Green Master Mix (TOYOBO). Data were analyzed with the comparative 2^ΔΔ^Ct method using the geometric mean of tubulin as a housekeeping gene. The primer sequences were as follows: *Stk4*, 5′-CATAAAGAGACTGGCCAGATT-3′ and 5′-GGCAGTTATTCCCAGAGACCA-3′; *Acta2*, 5′-TGCCGAGCGTGAGATTGTC-3′ and 5′-CGTTCGTTTCCAATGGTGATC-3′; and *Gsk3a*, qPCR Primer Pair (Beyotime; #QM08942S).

### Immunofluorescence

The LX-2 cells were fixed in 4% paraformaldehyde for 15 min at room temperature and then permeabilized with PBS containing 0.25% Triton X-100 for 10 min. After blocking with 5% BSA in PBS for 30 min, the cells were incubated with primary antibodies (anti-αSMA, Abcam, #ab240654; and anti-COL1A1, Cell Signaling, 1:1000 dilution, #72026) diluted in blocking solution, according to the manufacturer’s instructions, overnight at 4 °C. Cells were then incubated with Goat polyclonal Secondary Antibody to Mouse IgG H&L (Alexa Fluor 488; #ab150117) preabsorbed at 1:1000 dilution (shown in *green*) and Goat polyclonal Secondary Antibody to Rabbit IgG H&L (Alexa Fluor 594; #ab150080) at 1:1000 dilution (shown in *red*). Nuclear DNA was labeled with 4',6-diamidino-2-phenylindole (shown in *blue*) (Bytotime; #P0131). All photographs were taken using Nikon microscope.

### Proteomics Sample Preparation and LC–MS/MS Analysis

To achieve the deeper proteomics quantification, in-gel digestion was performed. Briefly, the samples were reduced and alkylated with 10 mM Tris(2-carboxyethyl)phosphine hydrochloride and 40 mM calmodulin for 20 min at room temperature and separated on 10% SDS-PAGE gel. Gels were stained in Coomassie Brilliant Blue solution containing 5% acetic acid and 20% ethanol. Coomassie Brilliant Blue was removed with destaining solution (30% acetonitrile [ACN]/35 mM ammonium bicarbonate, pH 7–8), dehydrated, solidified with ACN, and digested with trypsin (12.5 μg/ml in 50 mM ammonium bicarbonate) overnight at 37 °C. The tryptic peptides were then extracted from the gels with extraction buffer (5% formic acid [FA]/50% ACN) and centrifuged at 17,000*g*. Finally, the peptides were dried in a vacuum dryer.

Proteomics analysis using the data-dependent acquisition model was performed on a nano-UPLC chromatography system (Thermo Fisher Scientific) coupled to an ultraperformance liquid chromatography-Orbitrap Exploris 480 mass spectrometers. The resulting peptides were first dissolved in buffer containing 0.1% FA and 1% ACN and separated on a 75 μm I.D. × 20 cm capillary column (Beijing Spectra Peaks) packed with 1.9 μm C18 reverse-phase fused silica (Michrom Bioresources). Nonlinear LC separation was performed with an 80 min gradient from 8% to 40% mobile phase B (phase B: 0.1% FA in ACN, phase A: 0.1% FA/1% ACN in water) at a nanoflow rate of 300 nl/min. MS1 was then detected in the Orbitrap Analyzer using a wide scan (350–1500 *m/z*) with a resolution of 60,000 at 200 *m/z*. The normalized automatic gain control (AGC) was set at 300%, and the maximum injection time (MIT) was 50 ms. For MS2, the resolution was set at 15,000 with a normalized AGC target of 75% and an MIT of 80 ms. Dynamic exclusion was set to 45 s to suppress repeated detection of the same ion peaks. A normalized collision energy of 27% was used for precursor fragmentation in higher-energy collision dissociation mode.

### Phosphopeptide Enrichment and LC–MS/MS Analysis

Two milligrams of protein from each selected samples was digested with Lys-C (1:100, w/w) for 4 h, followed by trypsin (1:50, w/w) at 37 °C overnight. The resulting peptide mixture was acidified with FA (pH 2–3), loaded onto Sep-Pak tC18 cartridges (Waters), and desalted as follows: (a) methanol (MeOH) and ACN were used sequentially for pretreatment; (b) buffer B (80% ACN/0.1% FA) and buffer A (1% ACN/0.1% FA) were used to balance the column bed; (c) samples were loaded twice; (e) washing with buffer A; and (f) elution with 50% ACN/0.1% FA. The desalted samples were dried by vacuum centrifugation, and the peptides were frozen at −80 °C.

Phosphopeptide enrichment was performed using the High-Select Fe-NTA kit (Thermo Fisher Scientific) according to the manufacturer’s instructions. Briefly, the peptide samples were first dried and then dissolved in binding buffer (80% ACN and 0.1% TFA in ddH_2_O), the pH of which was adjusted to less than 3 using 0.1% TFA. The peptides and beads were mixed at a ratio of 1:1 (m/v) and incubated for 30 min at room temperature. The supernatant was discarded, and the samples were resuspended in 120 μl binding buffer and transferred to a filter tip. The samples were then slowly resuspended by adding 100 μl binding buffer (repeated four times) and ddH_2_O (repeated two times). Finally, the phosphopeptides were eluted twice with 100 μl elution buffer (5% NH_3_H_2_O). The eluates were collected and immediately dried in a SpeedVac concentrator at 45 °C and stored at −80 °C.

The resulting peptides were subjected to LC–MS/MS using an Orbitrap Fusion Lumos Mass Spectrometer (Thermo Fisher Scientific). Peptides were resolved in solvent A (0.1% FA and 1% ACN in ddH_2_O) and separated on a 150 μm I.D. × 20 cm capillary column packed with 1.9 μm C18 reverse-phase fused-silica (Michrom Bioresources). The LC nonlinear gradient with a 90 min ranges from 8% to 40% of mobile phase B (phase B, 0.1% FA in ACN; phase A, 0.1% FA/1% ACN in ddH_2_O) at a flow rate of 600 nl/min. Survey full scans were acquired from 300 to 1400 *m/z* at a resolution of 60,000 at 200 *m/z*, and the MIT was set to 50 ms or the AGC was set to 5 × 10^5^. The most intense precursors were selected for fragmentation per cycle with a dynamic exclusion time of 20 s. The activation type was higher-energy collision dissociation with normalized collision energy of 33%, the MS2 AGC was set at 1 × 10^4^, and the MIT was set at 35 ms.

### Targeted Detection of Phosphorylated Peptides of Kinases and Substrates

The relative abundances of phosphorylated substrates and kinases were detected by selective reaction monitoring (SRM) using the standard tier 3 level assay to monitor phosphorylated peptides (23). The detailed information of peptides for SRM is provided in supplemental Table S9. Following enrichment, the resulting peptide samples were dissolved in a sample buffer (1% FA and 1% ACN) and analyzed by the LTQ-Orbitrap Velos mass spectrometer in a survey scan (300–1600 *m/z,* resolution 30,000) followed by SRM scans for phosphopeptides in the LTQ. The intensities of the peptides were then analyzed by means of ion chromatograms using Xcalibur v2.0 software (Thermo Finnigan). Three biological repeats were applied in this study.

### Data Analysis

All raw files were searched with MaxQuant (version 2.1.4.0, Max Planck Institute of Biochemistry) against the Swiss-Prot reviewed mouse database (released on February 3, 2024, containing 17,201 entry proteins). Precursor mass tolerance was set to 20 ppm, allowing for tryptic cleavage with up to two missed cleavages. Fragment mass tolerance was set to 20 ppm. Carbamidomethylation of cysteine was set as a fixed modification, whereas oxidation of methionine and N-terminal acetylation of proteins were assigned as variable modifications. For phosphoproteome identification, phosphorylation of serine, threonine, and tyrosine residues (S, T, Y) were also set as variable modifications. The search results were filtered to a 1% false discovery rate at the protein, peptide, and peptide-spectrum match level using the target-decoy strategy. Peptide, protein, and phosphosite intensities were used for quantification.

Data analysis was primarily performed in Perseus (version 2.0.3.0, Max Planck Institute of Biochemistry) (24). The samples with missing values more than 33% (2/6) were filtered. Then, Log_2_-transformation and median normalization were performed. Two-sided unpaired Student’s *t* test was used for statistical analysis, and *p* < 0.05 was considered as statistically significant. Gene Ontology (GO) analysis and Kyoto Encyclopedia of Genes and Genomes pathway were performed using the online website Metascape (25) (http://metascape.org/) to obtain information about the biological processes involved in the differential proteins. Motif analysis of the selected phosphosites was validated using *p*Logo (https://plogo.uconn.edu/) (26). Predictive analysis of kinase substrates was validated using iGPS tool (https://igps.biocuckoo.org/) (27, 28). Interaction networks were constructed and mapped using Cytoscape and STRING analysis. Statistical analyses were performed with GraphPad Prism (version 10.1.2; GraphPad Software, Inc). Some graphs were created using an online data analysis and visualization platform (https://www.bioinformatics.com.cn and https://hiplot.com.cn/).

### Experimental Design and Statistical Rationale

For the establishment of CCl_4_-induced liver fibrosis mouse model and subsequent experiments, three biological replicates were used for CCl_4_ group and Oil group, respectively. For the pathological examination of the model, each mouse was stained with two slices as the technique was repeated. For the proteome and phosphoproteome of CCl_4_-induced mouse liver fibrosis, to ensure strict analysis, proteins quantified in more than 33% of samples or phosphosites quantified in more than 33% of samples with localization probability ≥0.75 were used for subsequent bioinformation analysis. For RT–qPCR analysis and all WB analyses, three biological replicates were performed. For SRM analysis of kinases and substrates, the intensities of peptides were analyzed by ion chromatograms using Xcalibur v2.0 software (Thermo Finnigan), and three biological repeats were applied. *P* Values less than 0.05 were significant and indicated by asterisks as follows: ∗*p* < 0.05, ∗∗*p* < 0.01, ∗∗∗*p* < 0.001, and ∗∗∗∗*p* < 0.0001.

## Results

### Early Stage Liver Fibrosis Mouse Model Was Induced by CCl_4_

In order to understand liver fibrosis more systematically and search for potential therapeutic targets for liver fibrosis, we performed proteomic and phosphoproteomic analyses of early mouse liver fibrosis samples. Mice were injected with CCl_4_, twice weekly, at repeated doses for up to 4 weeks to establish an early liver fibrosis model. This model captures important properties of hepatic oxidative fibrosis in a controlled manner, including inflammation, regeneration, and fibrotic scar formation (29). The mouse liver fibrosis model was evaluated by histopathological examination. H&E staining showed significant inflammatory cell infiltration around hepatocytes in the CCl_4_-treated liver (supplemental Fig. S1*A*). Sirius red staining showed that the characteristic architecture of the liver lobules was compromised by the infiltration of proliferative connective tissue in the CCl_4_-treated group (supplemental Fig. S1*B*). Blood biochemistry examinations were performed to further validate the model. The serum biomarkers alanine transaminase and aspartate transaminase were significantly increased in the CCl_4_-treated group as compared with the Oil group, which showed apparent toxicity in inducing inflammation (supplemental Fig. S1, *C* and *D*). Taken together, these results robustly confirm the successful establishment of an early stage mouse model of liver fibrosis characterized by pronounced inflammation, architectural distortion, and biochemical markers consistent with liver injury.

### Proteomic Analysis Revealed Dysregulation in Pathways Associated with ECM Deposition, Collagen Formation, and Several Metabolic Pathways

**Fig. 1:**
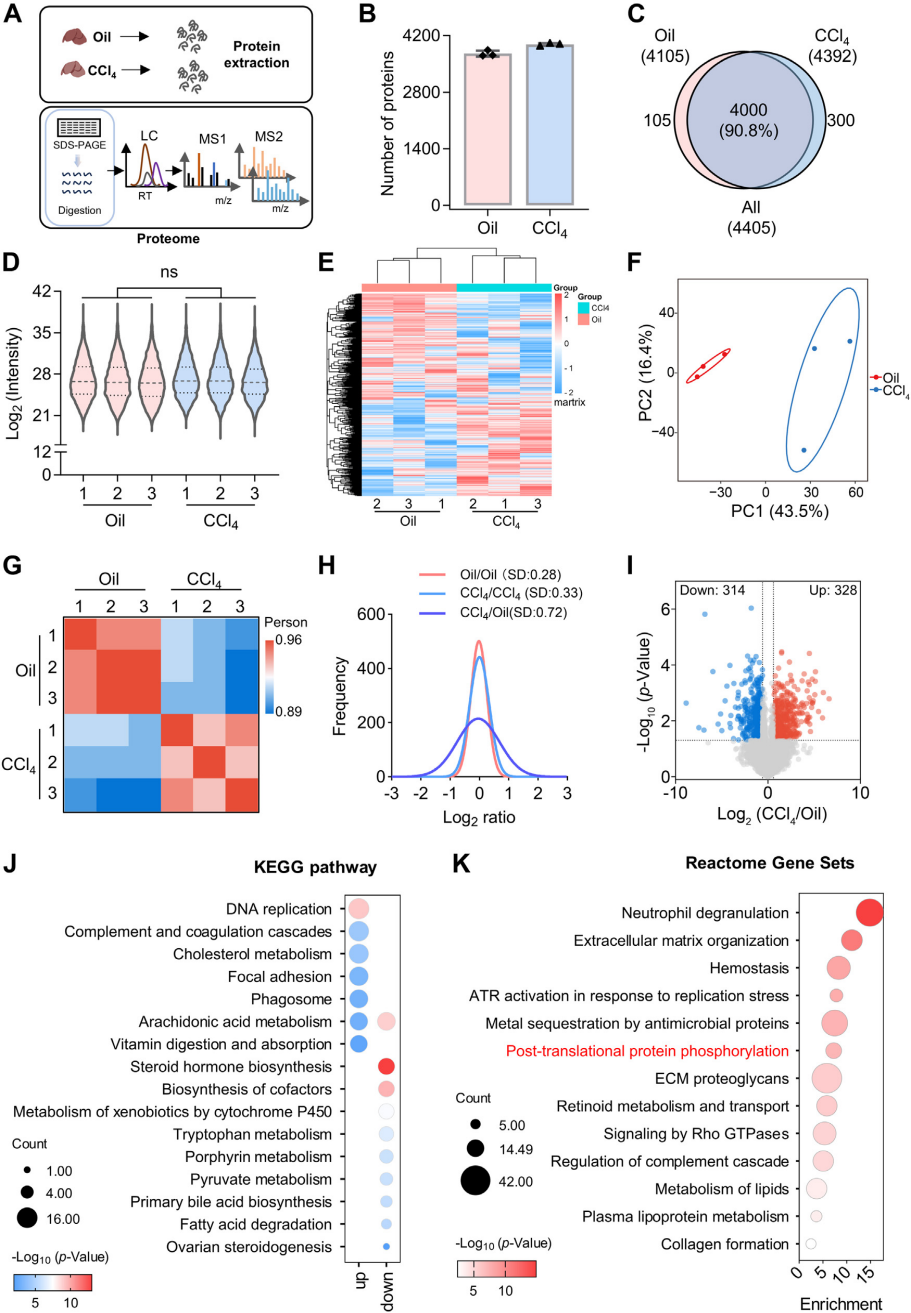
**Proteome profiling of mouse livers after CCl_4_-induced early liver fibrosis.***A*, workflow of proteomic analysis. *B*, the average number of proteins identified in proteome groups treated with Oil and CCl_4_. *C*, Venn diagram showed the proteins that overlapped between two groups. A total of 4405 proteins were identified in both groups, of which 4000 overlapped. *D*, the violin plot showed the overall distribution of the Log_2_-transformed intensity of the proteome datasets. There was no statistical difference between the two groups (ns, not significant). *E*, hierarchical clustering of the Oil- and CCl_4_-treated samples. *F*, principal component analysis (PCA) of Oil (*red*) and CCl_4_ (*blue*) groups. *G*, correlation coefficients of the two groups are presented in *red* (high positive correlation) and *blue* (low correlation). *H*, nonlinear regression fitting curve of the intensity ratio distribution within (Oil/Oil and CCl_4_/CCl_4_) and between (CCl_4_/Oil) groups. *I*, Volcano plot displayed the differently expressed proteins between the Oil and CCl_4_ groups based on a two-tailed Student’s *t* test. Significantly upregulated proteins (fold change ≥2 and *p* < 0.05) were colored *red*, and significant downregulated proteins (fold change ≤−2 and *p* < 0.05) were colored *blue*. *J* and *K*, KEGG pathway enrichment (*J*) and Reactome gene set enrichment (*K*). The size of the *circle* represents the number of genes involved, and the *color* indicates the significance of the enrichment. CCl_4_, carbon tetrachloride; KEGG, Kyoto Encyclopedia of Genes and Genomes.

To gain a comprehensive understanding of the global changes of liver fibrosis, we performed proteomic analysis of liver from mice treated with Oil and CCl_4_, respectively (Fig. 1*A*). First, whole tissue lysates of the equal mass were separated by SDS-PAGE (supplemental Fig. S2*A*) and digested with trypsin. Totally, more than 3700 and 3900 proteins were identified from three biological replicates of Oil- and CCl_4_-treated samples, respectively. A total of 4405 proteins were identified in the two conditions, of which 4000 proteins overlapped, accounting for 90.8% (Fig. 1, *B* and *C* and supplemental Table S1). The median of Log_2_ value of intensity was uniformly distributed across the proteome in both conditions, indicating no significant difference at the overall protein level (Fig. 1*D*). In addition, the clustering and principal component analysis of these two groups showed distinguishable features with each other (Fig. 1, *E* and *F*). The correlation between Oil groups was better than that between CCl_4_ groups (Fig. 1*G*). This is mainly because of the CCl_4_ compared with Oil treatment may cause more significant changes in the overall proteome and increase bigger heterogeneity.

Comparison of the Log_2_ fold change (FC) distribution among all groups showed that the median ratio of all groups was close to 0, but the SD of CCl_4_/Oil groups was 0.72, which was larger than that of Oil/Oil groups (0.28) or CCl_4_/CCl_4_ groups (0.33) (Fig. 1*H* and supplemental Fig. S2, *B*–*D*), indicating that the heterogeneity and significance were induced by the CCl_4_ stimulus. Based on this, 4308 proteins with two or more quantitative values were retained for subsequent differential screening. Among these proteins, 328 were upregulated in the CCl_4_ group (FC ≥2 and *p* < 0.05) and 314 were downregulated (FC ≤−2 and *p* < 0.05) (Fig. 1*I* and supplemental Table S2).

Functional enrichment analysis of the differentially expressed proteins showed that the upregulated proteins were mainly involved in ECM tissue, supramolecular fiber tissue, cell migration and adhesion, oxidative stress, and other biological processes highly related to liver fibrosis (supplemental Fig. S2*E* and supplemental Table S3). The downregulated proteins were mostly related to metabolic processes, such as monocarboxylic acid metabolism, carbon metabolism, and amino acid metabolism, which was consistent with literature reports (30, 31, 32, 33) (supplemental Fig. S2*F*). Consistent with the disorder in the GO_BP term, focal adhesion was also one of the most enriched Kyoto Encyclopedia of Genes and Genomes pathways of upregulated proteins, whereas cytochrome P450 metabolism and tryptophan metabolism were also the most significant pathways of downregulation (Fig. 1*J*). Furthermore, we found significant enrichment of protein phosphorylation in the processes involved in upregulated proteins, suggesting that post-translational modification of phosphorylation may play important roles in the progression of liver fibrosis (Fig. 1*K*).

### Phosphoproteomic Analysis Showed Significant Overall Increased Phosphorylation Signal in the Fibrosis Group

**Fig. 2:**
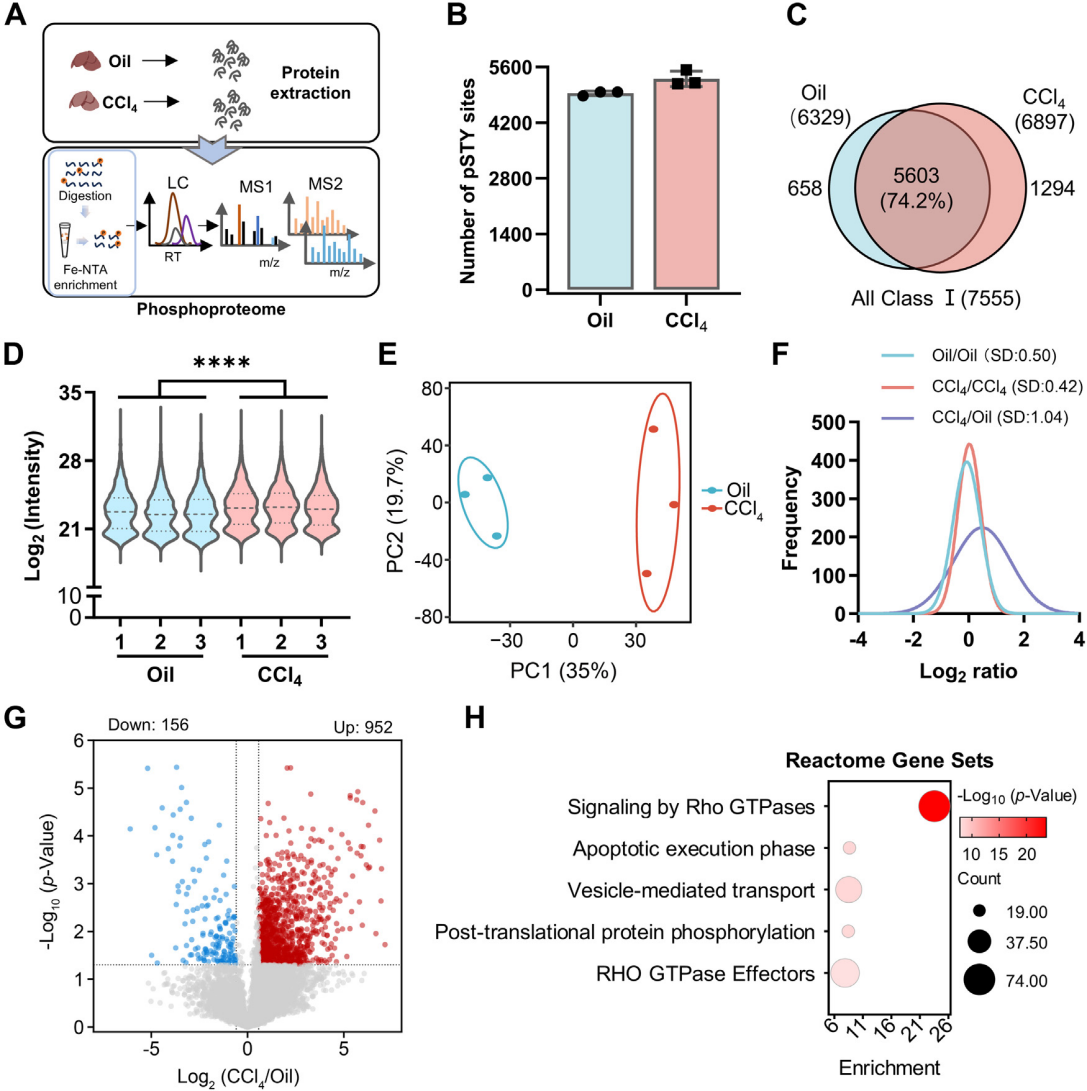
**Phosphoproteome profiling of mouse livers after CCl_4_-induced early liver fibrosis.***A*, workflow of phosphoproteomic analysis. *B*, the average number of class I phosphor-sites (STY) identified in phosphoproteome groups treated with Oil and CCl_4_. *C*, Venn diagram showed the sites that overlapped between two groups. A total of 7555 class I phosphosites were identified in both groups, of which 5603 overlapped. *D*, the violin plot showed the overall distribution of the Log_2_-transformed intensity of phosphosite datasets. There was significant difference between the two groups (∗∗∗∗*p* < 0.0001). *E*, PCA of Oil (*green*) and CCl_4_ (*red*) groups. *F*, nonlinear regression fitting curve of intensity ratio distribution within (Oil/Oil and CCl_4_/CCl_4_) and between (CCl_4_/Oil) groups. *G*, Volcano plot displayed the differently expressed phosphosites between the Oil and CCl_4_ groups based on a two-tailed Student’s *t* test. Significantly upregulated sites (fold change >1.5 and *p* < 0.05) were colored *red*, and significant downregulated proteins (fold change <−1.5 and *p* < 0.05) were colored *blue*. *H*, Reactome gene set enrichment analysis of upregulated phosphosites in *G*. The size of the *circle* represents the number of genes involved, and the *color* indicates the significance of the enrichment. CCl_4_, carbon tetrachloride; PCA, principal component analysis.

First, we observed that the overall phosphorylation level was upregulated after CCl_4_ treatment by immunoblotting (supplemental Fig. S3*A*). To further obtain an overall view of the abnormal phosphorylation, especially the upregulated ones, in liver fibrosis, phosphoproteomic analysis was performed (Fig. 2*A*). We totally identified 13,152 phosphorylation sites, of which 7555 (accounting for 57%) were class Ⅰ sites with a localization probability greater than 0.75 (supplemental Table S4). Of the 7555 phosphosites, 6203 (82.1%) were phosphoserine, 1161 (15.37%) were phosphothreonine, and 191 (2.53%) were phosphotyrosine, which is consistent with the general pattern of phosphorylation sites in mouse tissues (supplemental Fig. S3*B*) (34). The two groups identified an average of 5800 phosphorylated peptides and 4800 phosphorylated sites, of which 5603 phosphorylation sites overlapped, accounting for 74.2% (Fig. 2, *B* and *C* and supplemental Fig. S3*C*). Most phosphoproteins contained multiple sites, of which 68.7% were phosphorylated at two or more residues (supplemental Fig. S3*D*). The expression abundance and distribution of phosphorylation proteins in the control and model groups are intuitively shown by the violin plot. There were significant differences between the two groups (Fig. 2*D*). Principal component analysis showed a clear segregation among the Oil and CCl_4_ groups (Fig. 2*E*). Comparing the data distribution among the groups, it can be seen that the median Log_2_ ratio between the Oil/Oil group and the CCl_4_/CCl_4_ group was close to 0, but the CCl_4_/Oil group was significantly skewed to the right side to 0.49, and the SD value (1.04) was also significantly larger (Fig. 2*F* and supplemental Fig. S3, *E*–*G*), indicating the significant overall increased phosphorylation signal in the fibrosis group.

Based on this, 6302 sites with two or more quantitative values were retained for subsequent differential phosphosite screening. Of these, 952 were upregulated in the CCl_4_ group (FC >1.5 and *p* < 0.05), whereas only 156 were downregulated (FC <−1.5 and *p* < 0.05) (Fig. 2*G* and supplemental Table S5). The overall upregulation of phosphorylation levels in the aforementioned analysis was consistent with the WB results (supplemental Fig. S3*A*).

GO enrichment analysis (Fig. 2*H* and supplemental Table S6) revealed that the upregulated phosphosites were enriched in terms of cytoskeleton organization, regulation of GTPase activity, protein phosphorylation, an so on, whereas the downregulated phosphosites were enriched in lipid biosynthesis process and other biological processes (supplemental Fig. S3*H*). In addition, we observed significantly upregulated signaling of Rho GTPases, which is highly correlated with multiple biological pathways, such as cytoskeletal remodeling, cell migration and adhesion, and gene transcription (35, 36, 37).

### The Motifs Analysis With Significantly Upregulated Phosphorylation Levels Showed Obvious [xxxSPxxx] Characteristics

**Fig. 3:**
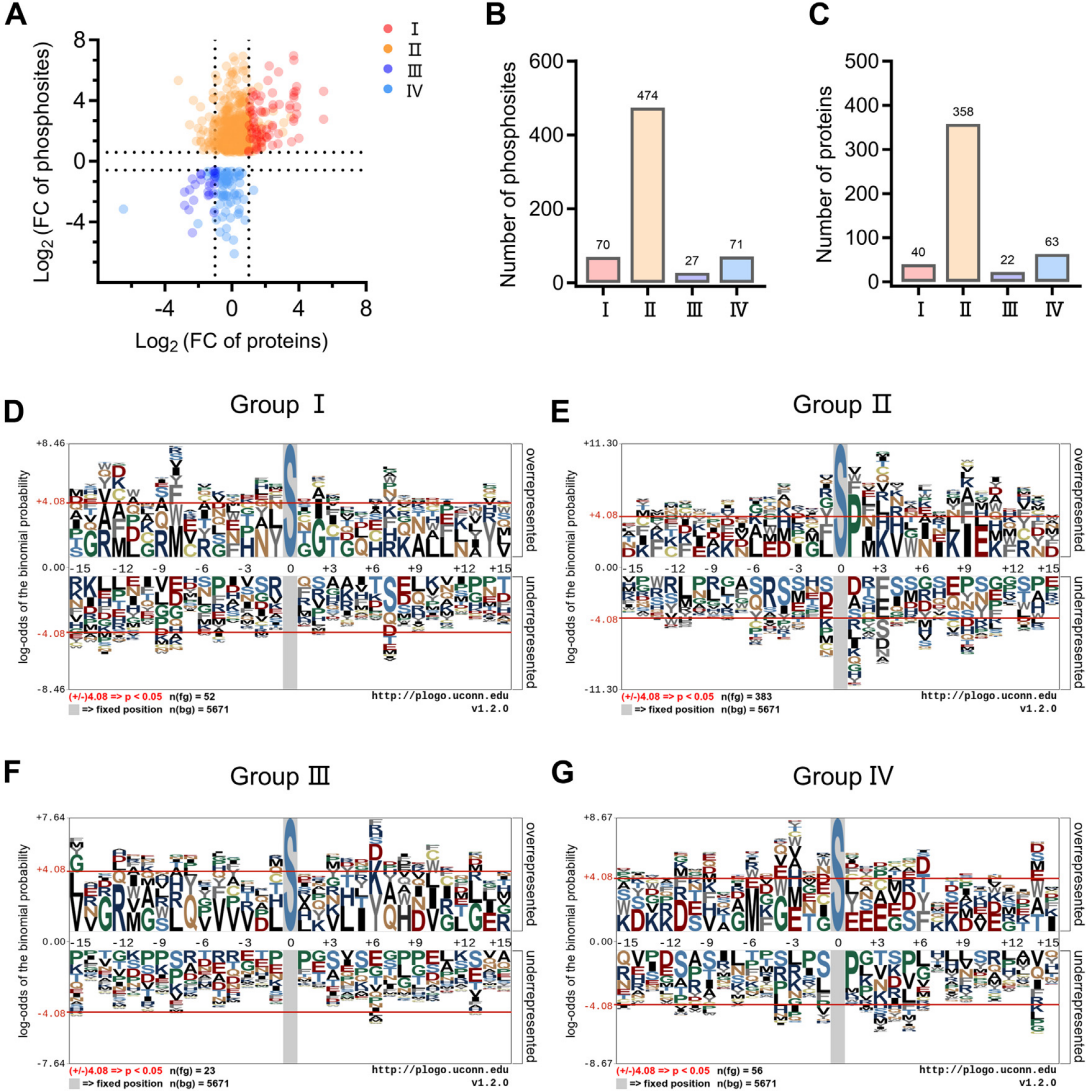
**Motif analysis of sites with significant changes.***A*, comparison of the changes of phosphosite (phos) abundance (fold change [FC] of phos) with those of the corresponding protein (pro) abundance (FC of pro). *Red* (Ⅰ) indicates that both phosphorylation and pro levels are significantly upregulated (*p* < 0.05, FC of phos >1.5, FC of pro ≥2). *Orange* (Ⅱ) indicates that the phosphorylation level was significantly upregulated, but the pro level was significantly downregulated or not changed (*p* < 0.05, FC of phos >1.5). *Blue* (Ⅲ) indicates that both phos and pro levels are significantly downregulated (*p* < 0.05, FC of phos <1.5 and FC of pro ≤−2). *Light blue* (Ⅳ) indicates that the phosphorylation level was significantly downregulated, but the pro level was not significantly changed (*p* [phos] <0.05, FC of phos <−1.5). *B* and *C*, number of phos (*B*) and pro (*C*) in *A*. *D*–*G*, motif analysis of group Ⅰ (*D*), group Ⅱ (*E*), group Ⅲ (*F*), and group Ⅳ (*G*).

To further investigate phosphorylation dysregulation events in liver fibrosis, we examined whether changes in differentially expressed phosphosites were due to changes in the protein about itself, changes in phosphorylation levels, or both. Changes in phosphosites were grouped into four categories: groups I and III were upregulated or downregulated at both phosphoproteomic and proteomic levels; group II was upregulated at phosphorylation levels only but unchanged and/or downregulated at protein levels; group IV was downregulated at phosphorylation levels only but unchanged at protein levels. It can be seen that the proportion of group II was the largest, with a total of 474 sites changed, corresponding to 358 phosphoproteins (Fig. 3, *A*–*C* and supplemental Table S7). To further evaluate the sequence characterization of the phosphorylated peptides, the phosphopeptide sequences with significant differences were analyzed using *p*Logo (26). Four groups of motifs were identified, predominantly comprising phosphoserine motifs. A total of 15 amino acid residues were identified on either side of each phosphorylation site (Fig. 3, *D*–*G*). The patterns of the four motifs exhibiting varying degrees of abundances, with group Ⅱ displaying a notable divergence from the other groups, exhibiting the sequence characteristics of [xxxSPxxx] (Fig. 3*E*). This indicates that this sequence may play a critical role in the response to liver fibrosis and could be employed as a potential targeted motif for the treatment of liver fibrosis. Furthermore, we utilized the iGPS platform (27, 28) and performed kinase–substrate prediction analysis. The prediction analysis was conducted on the sites and proteins of group Ⅱ, revealing that these kinases were mainly concentrated in the CMGC, CAMK, and STE families (supplemental Fig. S4, *A* and *B* and supplemental Table S8).

### Dysregulated Kinases Could be Used as Potential Targets for the Treatment of Liver Fibrosis

**Fig. 4:**
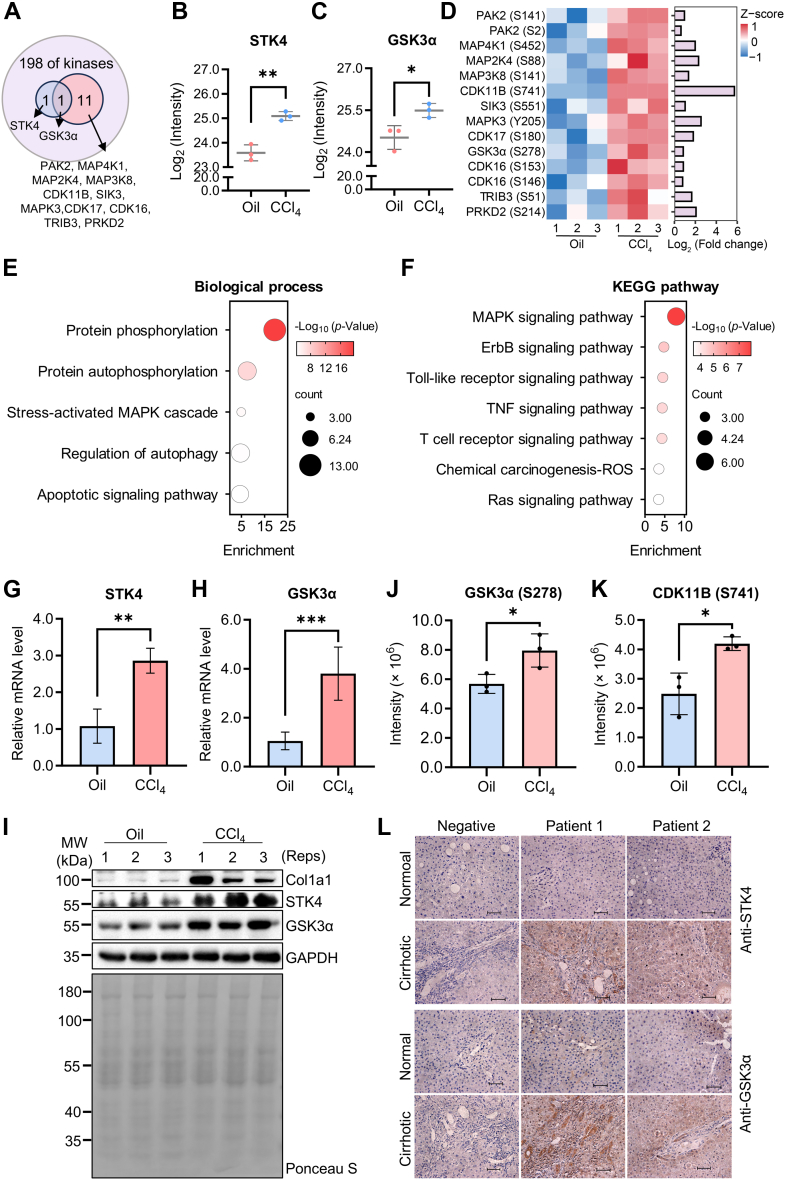
**Biological function analysis of identified upregulated kinases.***A*, the overlap between the predicted and upregulated identified kinases. Of the predicted 198 kinases, two were upregulated at the protein level, 12 were upregulated at the phosphorylation level, and one kinase overlapped. *B*, the expression of STK4 was upregulated at the proteome level by LC–MS/MS, ∗∗*p* < 0.01. *C*, the expression of GSK3α was upregulated at the proteome level by LC–MS/MS, ∗*p* < 0.05. *D*, heat map and bar charts of kinases with upregulated phosphorylation levels. The bar chart shows the Log_2_ (fold change) of kinases. *E*, biological process enrichment. *F*, KEGG pathway enrichment. The size of the *circle* represents the number of genes involved, and the *color* indicates the significance of the enrichment. *G* and *H*, relative mRNA level of STK4 (*G*) and GSK3α (*H*) in mouse livers after CCl_4_-induced early liver fibrosis was determined by RT–quantitative PCR, ∗∗∗*p* < 0.001, ∗∗*p* < 0.01. *I*, protein levels of STK4, GSK3α, and COL1A1 were determined by Western blotting. GAPDH serves as a loading control. *J*, the phosphorylated peptide, “GEPNVS(p)YICSR”, derived from GSK3α, was selected for quantification *via* selective reaction monitoring (SRM), ∗*p* < 0.05. *K*, the phosphorylated peptide, “RGTS(p)PRPPEGGLGYSQLGDDDLK”, derived from CDK11B was selected for quantification *via* SRM, ∗*p* < 0.05. *L*, the immunohistochemistry staining of STK4 and GSK3α in the liver tissues from two patients with liver cirrhosis. A negative control without antibody was selected. CCl_4_, carbon tetrachloride; GSK3α, glycogen synthase kinase 3α; KEGG, Kyoto Encyclopedia of Genes and Genomes; MS, mass spectrometry; STK4, serine/threonine-protein kinase 4.

A total of 198 kinases were predicted by our kinase–substrate prediction analysis using sites in group Ⅱ. Among these, 13 of which were also present in our upregulated proteomics or phosphoproteomics dataset (supplemental Table S8). Among them, two kinases were upregulated at protein level, whereas 12 kinases exhibited elevated phosphorylation level. Notably, one kinase exhibited both protein and phosphorylation level alterations (Fig. 4*A*). The upregulated kinases at the protein level were STK4, the major upstream kinase of the Hippo signaling pathway, which is involved in the regulation of cell proliferation, differentiation, and apoptosis (Fig. 4*B*). Another kinase that was upregulated at the protein and phosphorylation levels is GSK3α, a serine/threonine kinase that plays a key role in a multitude of biological processes, and its activity is regulated by autophosphorylation (Fig. 4, *A* and *C*). The remaining 11 kinases were found to be upregulated solely at the phosphorylation level within the analyzed dataset. The heat map showed the aforementioned kinases and their phosphorylation sites, some of which exhibited elevated phosphorylation levels at two sites (Fig. 4*D*). These dysregulated kinases were mainly involved in protein phosphorylation and autophosphorylation, autophagy regulation, and mitogen-activated protein kinase (MAPK) signaling, as well as other related signaling pathways (Fig. 4, *E* and *F*). Consequently, they may represent promising drug targets for liver fibrosis. Accordingly, three representative kinases from the upregulated dataset were selected for validation purposes. In the CCl_4_-induced group, STK4 and GSK3α exhibited a notable increase at both transcriptional and protein levels in comparison to the Oil groups (Fig. 4, *G*–*I*). Furthermore, SRM analysis revealed an elevated phosphorylation level of GSK3α and CDK11B, which were in line with the findings of the multiomics data analysis. In addition, upregulation of STK4 and GSK3α was also observed in the liver cirrhosis patients by immunohistochemistry staining (Fig. 4*L*).

**Fig. 5:**
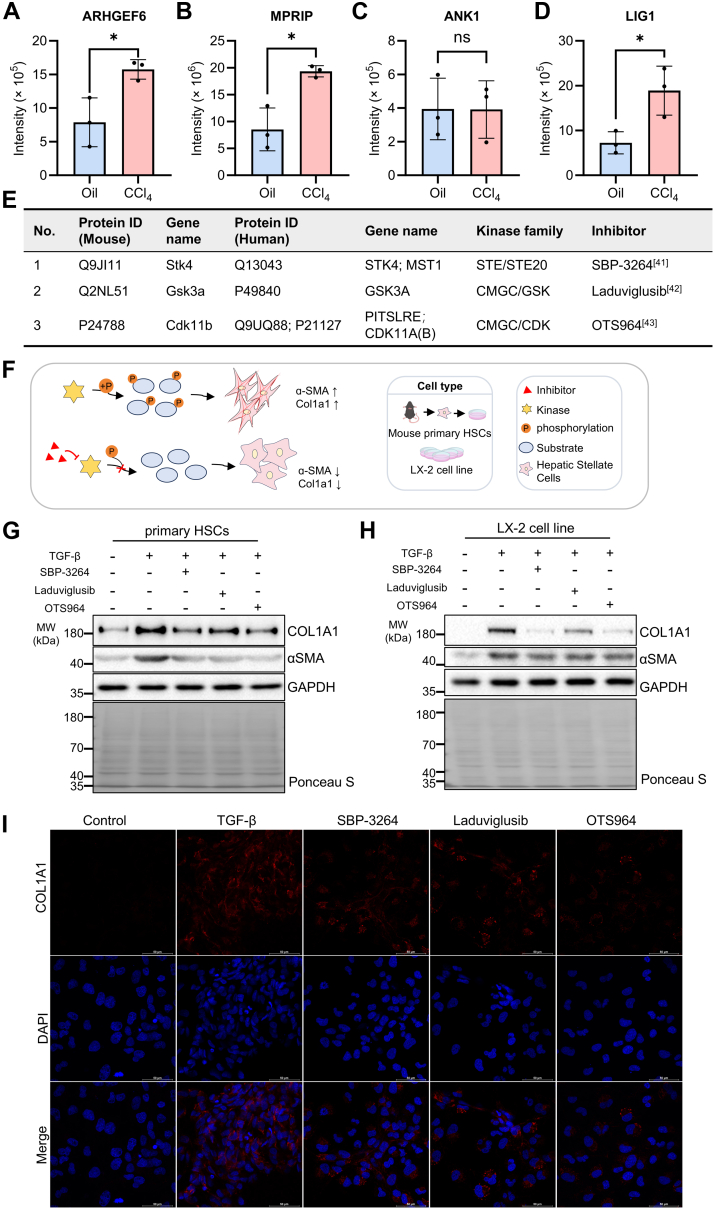
**Inhibition of kinase activity by target inhibitors can reduce hepatic stellate cell (HSC) activation.***A*–*D*, the downstream substrate phosphorylation levels of three kinases in the liver of CCl_4_ mice were monitored by SRM targeting. Detailed information about the phosphopeptides monitored by SRM can be found in supplemental Table S9. Phosphorylation levels of ARHGEF6 (*A*), a substrate of STK4, were upregulated after treatment with CCl_4_; MPRIP (*B*) and ANK1 (*C*) are substrates of GSK3α. The phosphorylation level of MPRIP was upregulated after treatment with CCl_4_, whereas the ANK1 remained unchanged; phosphorylation levels of LIG1 (*D*), a substrate of CDK11B, were upregulated after induced by CCl_4_. ∗*p* < 0.05; ns, not significant. *E*, basic information of three kinases (in *Mus musculus* and *Homo sapiens*) and their reported small-molecule inhibitors. *F*, experimental design to validate the antihepatic fibrosis potential of small-molecule inhibitors targeting kinases. *G* and *H*, the expression of COL1A1 and αSMA was determined by Western blotting after treated with 10 ng/ml TGF-β1 alone or along with kinase inhibitors (200 nM STK4 inhibitor SBP-3264; 100 nM GSK3α inhibitor laduviglusib; 100 nM CDK11B inhibitor OTS964) in mouse primary HSCs (*G*) and LX-2 cell line (*H*). *I*, immunofluorescence showed that TGF-β stimulation could significantly inhibit the activation of HSCs by adding kinase inhibitors, and the production of COL1A1 was reduced. CCl_4_, carbon tetrachloride; GSK3α, glycogen synthase kinase 3α; SRM, selective reaction monitoring; TGF-β1, transforming growth factor beta-1.

Furthermore, an interaction network diagram was constructed for these 13 kinases and their active substrates (supplemental Fig. S5*A*). The interaction between kinases and substrates reflects the complex nature of the pathological process underlying liver fibrosis. Upregulation of kinases has been observed to increase the phosphorylation expression of downstream substrates (Fig. 5, *A*–*D* and supplemental Table S9), which may be critical in causing activation of downstream pathways and inducing progression of liver fibrosis. Our study clearly showed that the abnormal kinase activity was closely associated with the occurrence and progression of liver fibrosis.

At present, research on therapeutic strategies for liver fibrosis primarily focus on targeted inhibition of kinases, which has the potential to alleviate liver fibrosis by inhibiting kinase activity. Small-molecule kinase inhibitors can target kinases and inhibit cell proliferation and angiogenesis, thereby playing an antifibrosis role and representing a promising class of therapeutic agents for the treatment of hepatic fibrosis (14, 38, 39). Through omics analysis, we identified 13 kinases that may play an important role in the development and progression of liver fibrosis. These 13 kinases belong to the STE, CMGC, and CAMK families, respectively (supplemental Fig. S5*B*). Consistent with the majority of existing literature, the [xxxSPxxx] motifs are identified as typical proline-directed motifs with the potential to serve as substrates for the CMGC kinase family, including MAPK, CDK, and CDK-like (28, 40, 41, 42). This indicated that the kinase that acts selectively on the [xxxSPxxx] sequence may represent potential targets for the treatment of liver fibrosis, and that inhibitors of such kinases may constitute a potential class of antihepatic fibrosis drugs.

### Inhibition of Kinase Activity by Target Inhibitors Can Reduce the Activation of HSCs

To validate the hypothesis, a further literature survey was conducted with the objective of finding some of the reported useful kinase inhibitors (supplemental Fig. S5*B*). Finally, we selected inhibitors of three kinases for cell model validation: SBP-3264 for STK4 (43), laduviglusib for GSK3α (44) and OTS964 for CDK11B (45) (Fig. 5*E*). The mouse primary HSCs and human LX-2 cell line were treated with TGF-β alone or combined with small-molecule inhibitors of three kinases (Fig. 5*F*), respectively. The results demonstrated that the expression levels of both αSMA and COL1A1, which are important indicators of HSC activation, were diminished in the activated cells following inhibitor treatment (Fig. 5, *G* and *H*). These findings indicated that kinase inhibitors could effectively delay the progression of liver fibrosis. Similarly, we demonstrated by immunofluorescence that kinase inhibitors can reduce the activation of LX-2 cells, and the expression of αSMA and collagen I was reduced to varying degrees (Fig. 5*I* and supplemental Fig. S6*A*). However, the effect of different kinase inhibitors was not uniform. Of the three inhibitors, the effect of the GSK3α inhibitor was slightly less pronounced than that of the other two inhibitors, which may be due to the difference in the optimal inhibitory concentration of the different inhibitors. At the same time, differences were observed between mouse primary cells and human cell lines, indicating that phosphorylation events in cell lines may be more complex and exhibit a dose dependence in inhibitors. Therefore, we treated the cells with varying concentrations of the inhibitors and demonstrated that, consistent with our hypothesis, the levels of collagen I decreased in a dose-dependent manner as the concentration of the drug increased (supplemental Fig. S6, *B*–*D*). In additionally, the downstream pathway activated by kinases may be associated with the occurrence and progression of liver fibrosis, which deserves to be explored in depth.

### Kinase Inhibitors Could Reduce the Phenotype of Early Liver Fibrosis in Mice

**Fig. 6:**
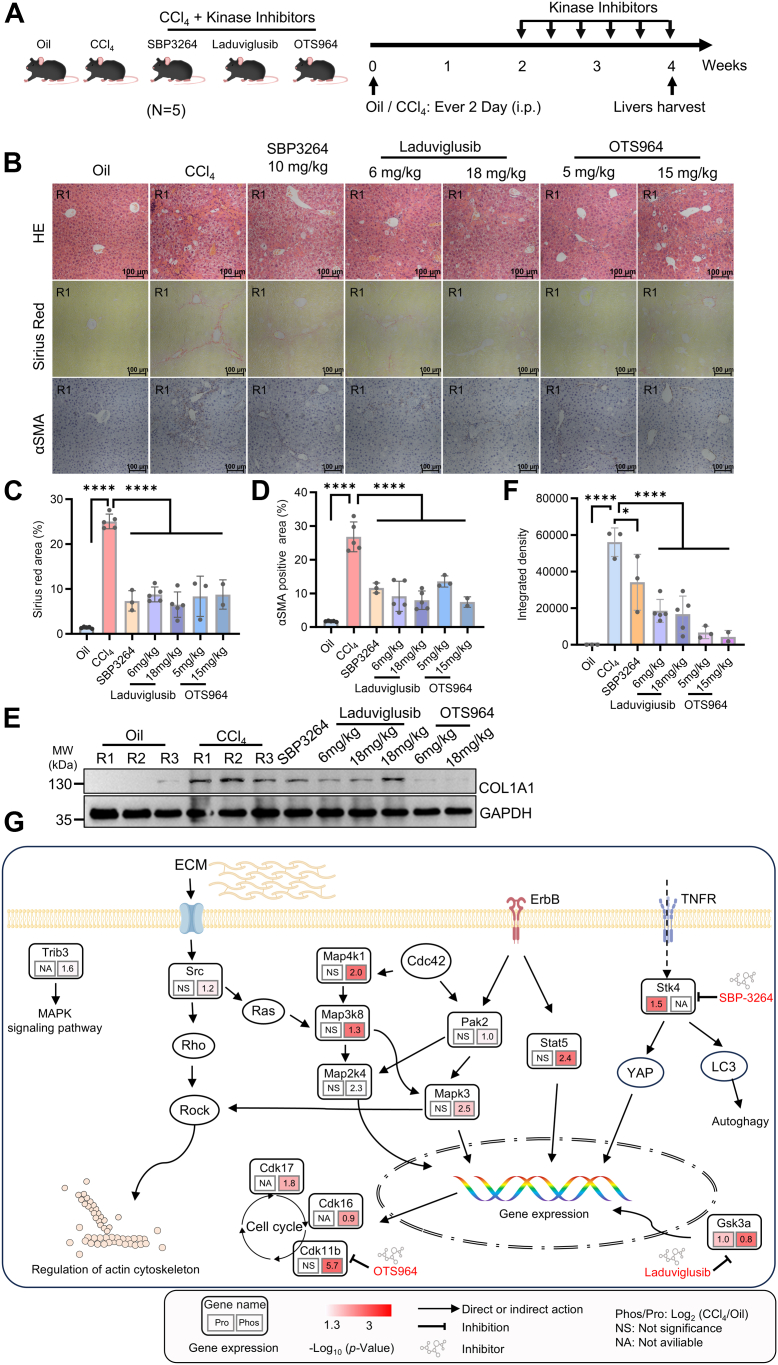
**The use of kinase inhibitors could reduce the phenotype of early liver fibrosis in mice.***A*, workflow of kinase inhibitors (SBP-3264, 10 mg/kg; laduviglusib, 6 mg/kg or 18 mg/kg; and OTS964, 5 mg/kg or 15 mg/kg) treatment in CCl_4_-induced fibrotic mice model (n = 5 in each group). *B*–*D*, liver tissues from vehicle- or inhibitor-treated fibrotic mice were stained with H&E, Sirius *Red*, and α-SMA, (*B*) and quantitative results of Sirius *Red* (*C*) and αSMA staining (*D*) are shown. More results can be available at supplemental Figs. S7–S9. ∗*p* < 0.05, ∗∗∗∗*p* < 0.0001. *E*, WB analysis of COL1A1 in the liver after treatment with kinase inhibitors. Due to the limited number of samples on the SDS-PAGE, only some of the results from each group are shown in this panel, and more results are available in the supplementary Figure S10. *F*, quantification of all lanes from *E* and Figure S10, the *gray* values of the same group are combined into one column. *G*, biological landscape of partially upregulated proteins and kinases in an early hepatic fibrosis model. *Red*, *p* < 0.05; *white*, not significant or not available. The fold change of genes enriched was shown. Small-molecule inhibitors of kinases are highlighted in *red*. CCl_4_, carbon tetrachloride; WB, Western blot.

By targeting specific signaling pathways, drugs can interfere with cellular activities that lead to fibrosis, such as reducing stellate cell activation and proliferation, reducing inflammation, and promoting the degradation of already deposited ECM (18, 19, 20, 39, 46). Kinase inhibitors have shown potential in treating liver fibrosis in mice. To further verify whether these kinase inhibitors can alleviate liver fibrosis in mice, a 4-week study was conducted (Fig. 6*A*). During the initial 2 weeks, all mice received undifferentiated intraperitoneal injections of CCl_4_ to adequately induce liver fibrosis. This step ensures that all animals develop a consistent level of fibrosis, providing a reliable baseline for subsequent treatments. After 2 weeks, the mice were randomly assigned to different treatment groups and injected with corresponding kinase inhibitors. Each group consisted of five mice to ensure data reliability and reproducibility. Specifically, for both GSK3α and CDK11B, two dose levels of inhibitors were tested (Fig. 6*A*). This approach not only helps in assessing the effectiveness of the inhibitors but also aids in determining the optimal dosage for therapeutic use. Histologic analysis demonstrated that treatment with kinase inhibitors effectively suppressed HSC transdifferentiation, collagen production, and fibrotic septa formation in CCl_4_-challenged liver tissues (Fig. 6, *B*–*E*, supplemental Figs. S7–S9 and S10, *A*–*C*). In order to further verify the changes in kinase phosphorylation levels after the addition of kinase inhibitors, we selectively detected the phosphorylation levels in the high-dose group of GSK3α inhibitor laduviglusib. The results showed that the phosphorylation level of GSK3α was significantly reduced after the addition of inhibitors (supplemental Fig. S10*D*), suggesting that the phosphorylation level of kinase affects the kinase activity and plays an important role in the process of liver fibrosis in mice.

While the findings in mouse models provide encouraging evidence for the potential of kinase inhibitors in treating liver fibrosis, the study also revealed several key issues and challenges: Not all mice tested showed effective reduction in liver fibrosis symptoms after treatment with kinase inhibitors, indicating significant individual variability (supplemental Figs. S7–S10). This difference may be related to genetic background, stage of disease progression, or varying responses to drugs. During the administration of two inhibitors, SBP3264 and OTS964, death was observed in some mice. This phenomenon suggests that these inhibitors might have toxicity or serious side effects. Further research is needed to delve into the specific causes of death, including but not limited to drug dosage, metabolic effects, and impacts on nontarget tissues. Most mice experienced weight loss before and after treatment, which could be due to drug-induced appetite loss, metabolic changes, or other health problems. This highlights the need to consider both the safety of the drug and its impact on the overall health status of the animal when evaluating treatment effectiveness. Future studies should focus on optimizing the dosing regimen to determine the optimal dose range that effectively alleviates liver fibrosis without causing significant side effects. In light of the significant disparities between animal models and human clinical applications, further researches are imperative to substantiate the safety and efficacy of these inhibitors in clinical researches. While the preliminary outcomes are encouraging, it is crucial to address these concerns through further research to develop safe and effective treatments for liver fibrosis. Elucidation of the mechanisms underlying these observations will also furnish valuable insights for the treatment of other related diseases.

To show the function of kinases in cells in a more intuitive way, we have provided a comprehensive graphical representation of the entire process. These kinases can further activate downstream pathways by increasing their own levels or their own phosphorylation levels. Moreover, some kinases, such as TRIB3, can also act as upstream regulators of the entire signaling pathway. It is not difficult to see that the ultimate biological effects of these kinases are mainly actin skeleton regulation, cell cycle circulation, and gene expression (Fig. 6*G*), which are closely related to the physiological and pathological mechanisms of liver fibrosis.

The findings of our study indicate that the total protein phosphorylation levels in the liver are predominantly upregulated at the early stage of liver fibrosis progression, and the upregulated phosphosites exhibited a common phosphorylation motif [xxxSPxxx]. This suggests that kinases that act on the [xxxSPxxx] sequence may be potential targets for the treatment of liver fibrosis, and that inhibitors of these kinases may be potential drugs against liver fibrosis. To test this hypothesis, three kinase inhibitors were selected for validation in mouse primary cells and human HSC lines. As anticipated, the addition of kinase inhibitors inhibited the activation of HSCs and decreased the expression of αSMA and COL1A1. Kinase inhibitors could also reduce the symptoms of CCl_4_-induced liver fibrosis in mice. Although not all tested inhibitors demonstrated equal efficacy, and some inhibitors (such as SBP3264 and OTS964) exhibited potential toxicity or side effects, these results collectively provide a foundation for developing safer and more effective treatments.

## Discussion

It is very important to comprehensively understand the pathological development of liver fibrosis and search for potential targets for intervention and treatment of liver fibrosis (47). To date, MS-based omics research has developed rapidly and made great contributions to exploring the pathological mechanism of diseases (48). Phosphoproteomics has been employed as a means of identifying potential therapeutic targets. On this basis, multiomics studies combined with proteomics are more classic, because they not only screen for differentially expressed phosphorylated proteins but also provide more information about whether these differences are due to changes in the protein itself, changes in the phosphorylation level, or changes in both levels. Up to now, the joint analysis of phosphoproteomics with global proteomics is little (49). Therefore, we performed proteomic and phosphoproteomic analyses to further reveal the role of phosphorylation in the development of liver fibrosis and to explore potential therapeutic targets for the treatment of liver fibrosis.

Our study screened 13 changed kinases mainly belonging to the STE, CMGC, and CAMK families. Consistent with most studies in this field, CMGC kinases, particularly CDKs and MAPKs, can specifically recognize substrates containing SP sequences. Most kinases can regulate their activity by changing their own content or phosphorylation level, which further affects downstream signaling pathways (50, 51, 52).

Studies have shown that STK4 and TRIB3 further participate in the occurrence and development of liver fibrosis by regulating the level of autophagy (53, 54). Although the role of autophagy in promoting or inhibiting liver fibrosis is still controversial, autophagy is still a potential target for the treatment of liver fibrosis (55, 56). Moreover, STK4 is a key component of the Hippo signaling pathway, regulating cell proliferation and death (57). In mammalian cells, Hippo signaling can be specifically inhibited by the use of compound 20 (SBP-3264), which in turn inhibits cell proliferation (43). Furthermore, the process of liver fibrosis is characterized by the proliferation of a large number of HSCs, resulting in a significant increase in cell number. This indicates abnormal cell cycle regulation, which is accomplished through a series of phosphorylation and dephosphorylation reactions of CDKs (58, 59). In our dataset, we observed an increase in the phosphorylation levels of CDK11B, CDK16, and CDK17. Previous studies have shown that the use of the CDK inhibitor roscovitine can reduce liver inflammation and fibrosis (60). This suggests that these kinases may serve as potential targets for the treatment of liver fibrosis. In addition, GSK3α plays an important role in liver glucose metabolism, participates in the Wnt signaling pathway, apoptosis, and other biological processes. The GSK3α is a highly important kinase in liver and can be used to treat type 2 diabetes by inhibiting kinase activity (44, 61). The aforementioned evidences further suggest that these kinases play an important role in the physiological and pathological processes of the liver and may be used as potential therapeutic targets.

The use of small-molecule inhibitors to target kinases and inhibit cell proliferation represents a promising method for the treatment of liver fibrosis (20, 46, 62). At present, positive therapeutic effects of certain small-molecule inhibitors have been observed in preclinical animal models of liver fibrosis and in patients with liver fibrosis (63, 64). The present study identified multiple potential kinase targets for antihepatic fibrosis through multiomics data analysis and preliminarily verified the antihepatic fibrosis effects of three kinase targets and their small-molecule inhibitors though cellular and animal’s experiments. The findings of this study may provide a broader perspective for the treatment of liver fibrosis and offer further evidence in support of targeted kinase therapy.

Nevertheless, it must be acknowledged that the dataset is not without flaws. First, the sample size of the data is insufficient for achieving a deeper and broader level of identification. And a limitation of the current study is that our whole proteome and phosphoproteome comparisons were based on entire liver tissue samples. The cellular composition of the liver is highly complex, including but not limited to hepatocytes, HSCs, and immune cells. Consequently, the results of this study reflect a composite signal from multiple cell types rather than specific changes in a single cell type. Not withstanding this limitation, some interesting and significant changes in the mixed samples were observed, which provide valuable clues for further investigation. Second, although the therapeutic effect of kinase inhibitors has been preliminarily verified, the specific mechanism has not been deeply explored and confirmed. Notably, TGF-β stimulation of LX-2 cells did not result in changes in the protein level of STK4 and GSK3α and may not cause an increase in the phosphorylation levels of GSK3α and CDK11B. This finding suggests that the activation of kinases, such as STK4, GSK3α, and CDK11B, or their response to TGF-β stimulation, might not originate directly from HSCs themselves but could be influenced by other cell types. *In vivo*, the functionality of these kinases and their regulatory impact on targets such as collagen Ⅰ are likely to occur through intercellular interactions or signaling pathways. Consequently, when investigating the mechanisms of action for these kinases, it is crucial to consider their sources and the dynamics of their interactions across various cell types. Furthermore, this study utilized a single period of model mice, and the kinase disturbance events at different periods of liver fibrosis remain to be elucidated. At the same time, we did not conduct more clinical sample validation. We recognize that further analysis of the obtained mouse liver samples from these experiments might provide valuable insights into the potential mechanisms of liver fibrosis and dysregulated kinases. It is hoped that in the future, we can continue to explore the kinase target therapy of liver fibrosis and provide strong evidence for the treatment of liver fibrosis.

## Data Availability

The MS proteomics data have been deposited to the ProteomeXchange Consortium (https://proteomecentral.proteomexchange.org) *via* the iProX partner repository (65, 66) with the dataset identifier PXD059906 or IPX0008780000. Annotated spectral files were uploaded to the MS-viewer (67). The search key for proteome is *ata9y6hctj.* The search key for the phosphorated peptides is *rlz4mgowhw*.

## Supplemental data

This article contains supplemental data, including 10 supplemental figures and 9 tables (43, 44, 68, 69, 70, 71, 72, 73, 74, 75, 76, 77, 78).

## Conflict of interest

The authors declare no competing interests.
