# Supplementary material for: Proteomics and Phosphoproteomics Revealed Dysregulated Kinases and Potential Therapy for Liver Fibrosis
Source: Mol Cell Proteomics. 2025 May 12;24(6):100991. doi: 10.1016/j.mcpro.2025.100991 (PMC12181035; doi:10.1016/j.mcpro.2025.100991)
Supplement: Supplemental data [file mmc10.docx]

**Supplemental data**


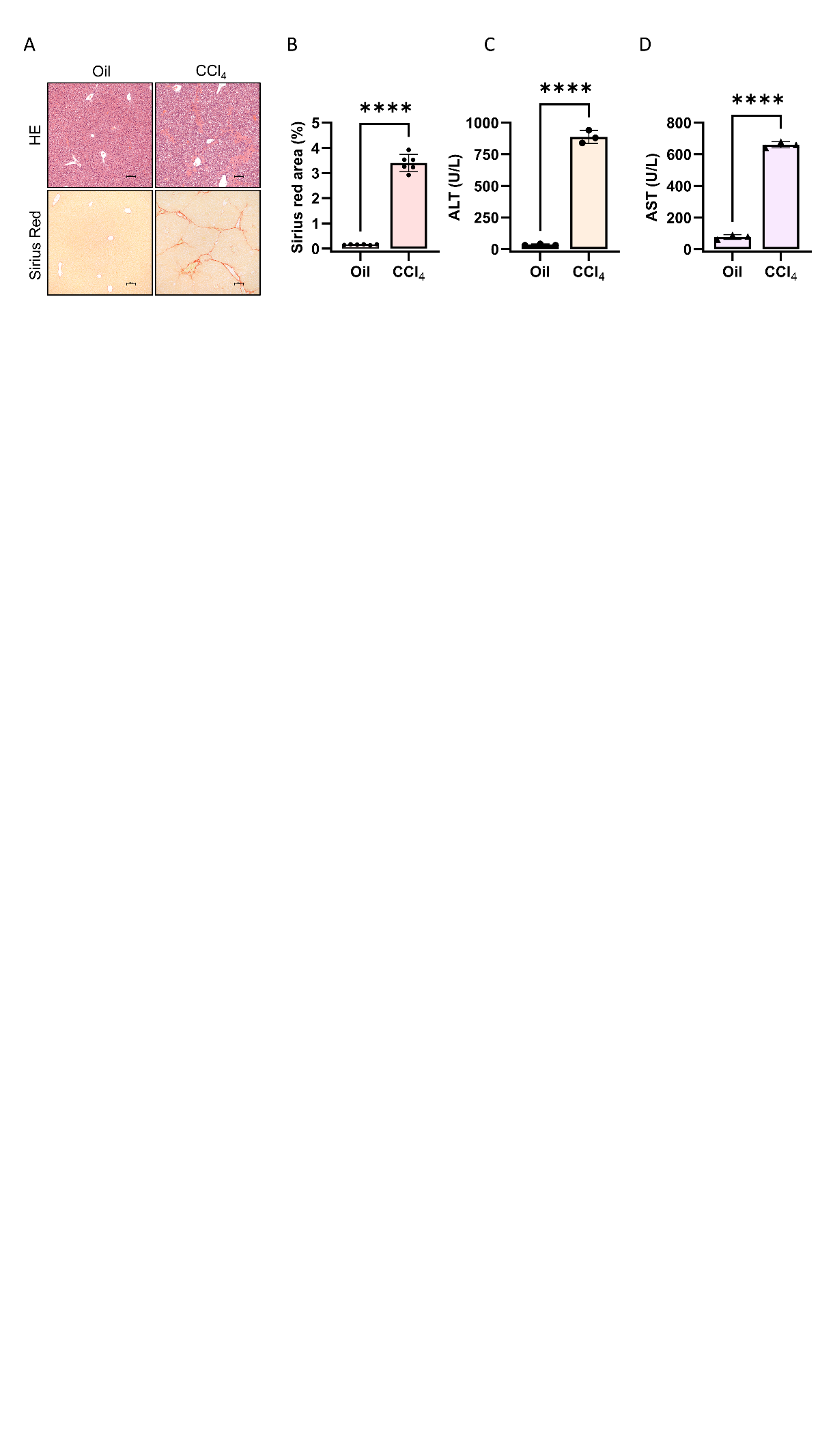


Figure S1. Successful establishment of CCl_4_-induced early liver fibrosis model. (A) Hematoxylin-eosin (HE) staining and Sirius red staining. Parenchymal destruction was observed in HE staining and collagen fibers appear in red in Sirius red staining of CCl_4_-induced liver fibrosis livers. Scale bar corresponds to 100 μm. (B) Quantitative results of the area with positive scarlet staining of Sirius. (C) Serum ALT level. (D) Serum AST level. Data are presented as mean ± SD (n = 3). **** *p*-Value < 0.0001, compared with CCl_4_ group. Oil, control group; CCl_4_, carbon tetrachloride treated group.


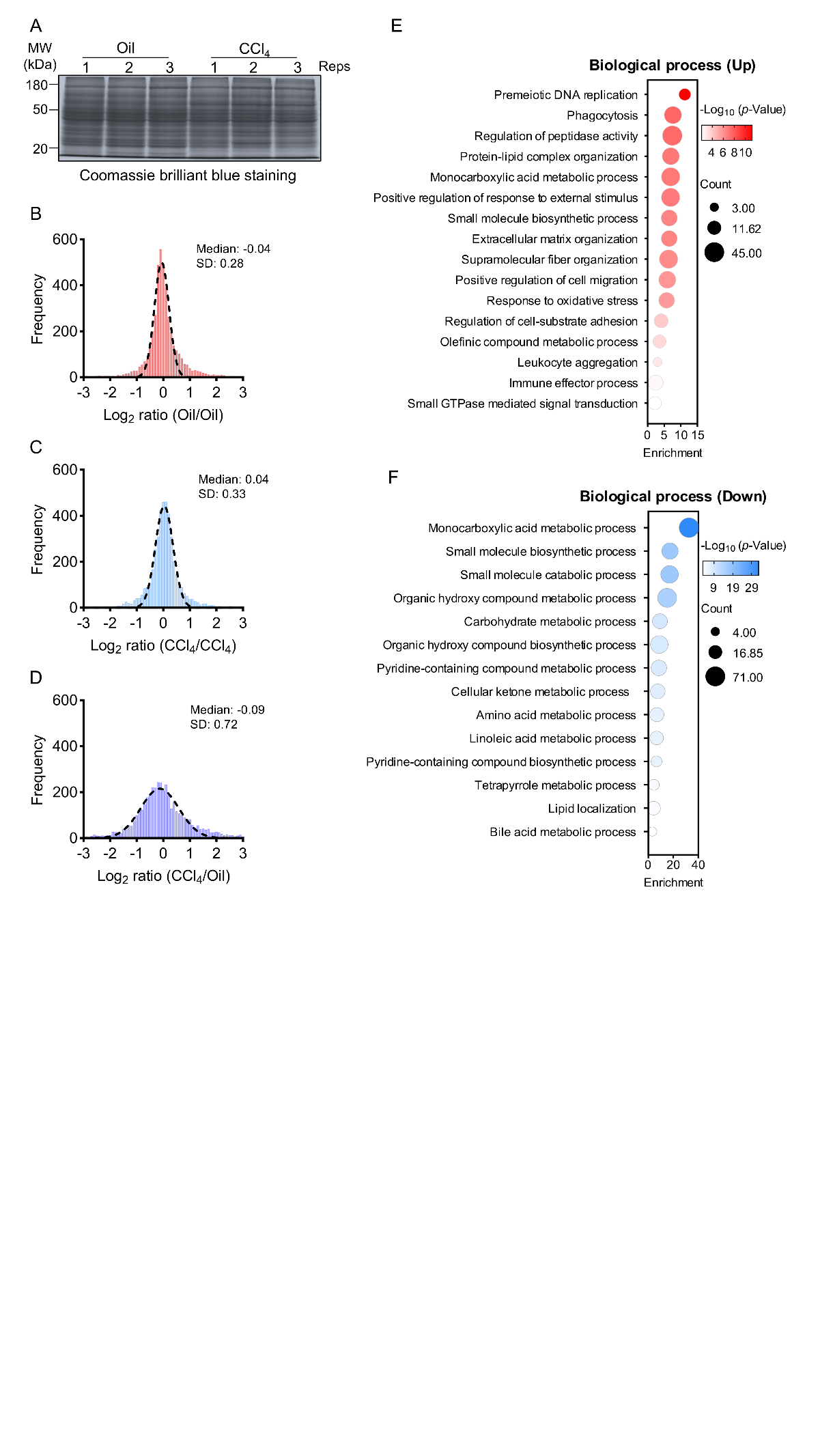


Figure S2. FC ratios distribution and Gene Ontology (GO) analysis of differentially expressed proteins detected by proteomic analysis. (A) Coomassie bright blue staining of liver whole tissue lysates separated by SDS-PAGE. (B) & (C) Frequency histogram of intensity ratio distribution within groups, Oil/Oil (B) and CCl_4_/CCl_4_ (C), the dashed lines are Nonlinear regression fit curves. (D) Frequency histogram of intensity ratio distribution between groups (CCl_4_/Oil), the dashed lines are Nonlinear regression fit curves. (E) & (F) Biological process of GO enrichment analysis of differentially expressed proteins in Figure 1I. The size of the circle represents the number of genes involved, and the color indicates the significance of the enrichment.


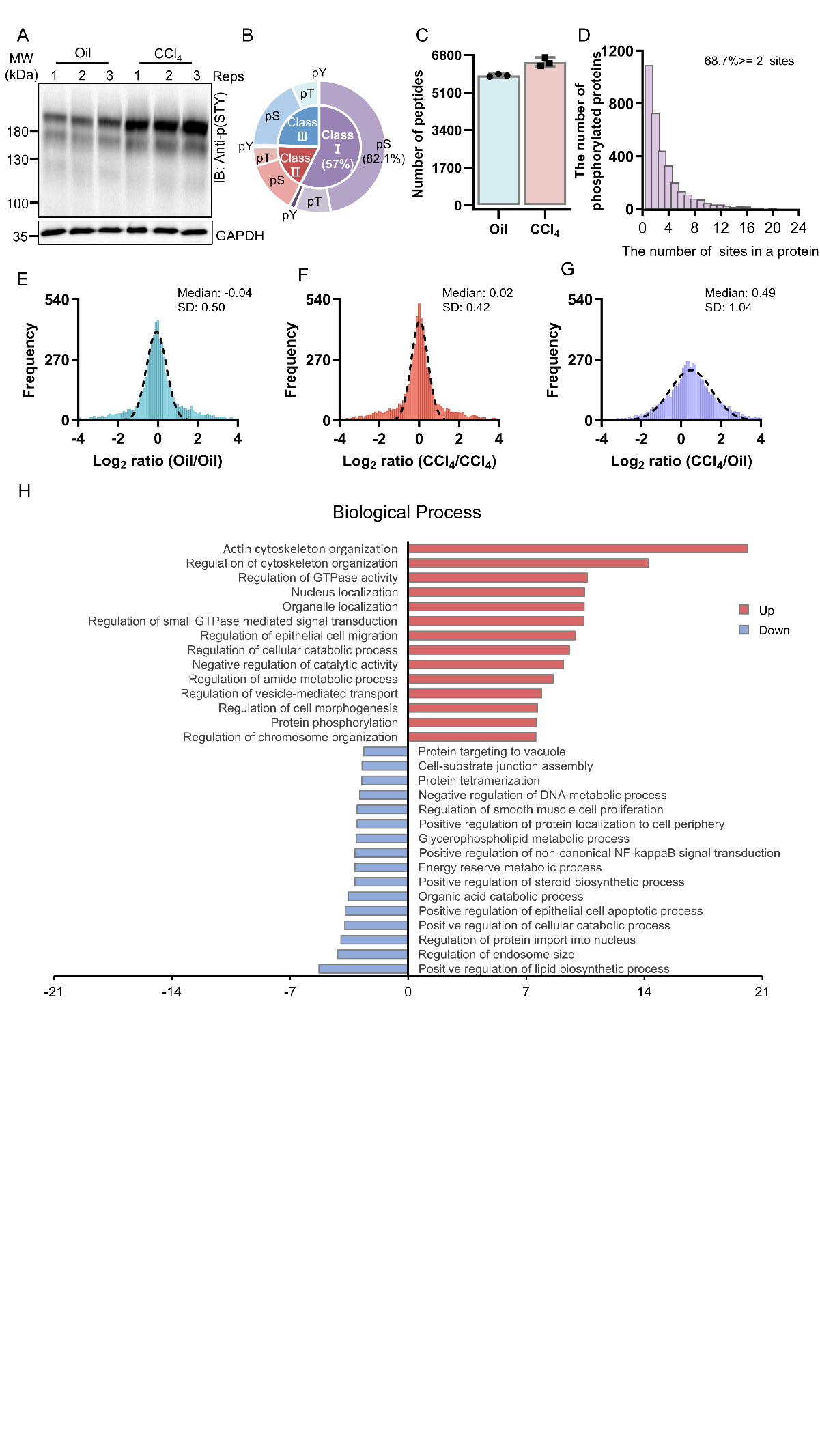


Figure S3. Phosphoproteome profiling of mouse livers after CCl_4_-induced early liver fibrosis. (A) Immunoblotting analysis of phosphorylation signal changes after CCl_4_ treatment, with GAPDH as the loading control. (B) The proportion of the type of phosphorylation sites and pS, pT, pY sites. (C) The average number of peptides identified in phosphoproteome groups treated with Oil and CCl_4_. (D) Histogram depicting numbers of sites observed per protein. (E) & (F) Frequency histogram of intensity ratio distribution within groups, Oil/Oil (E) and CCl_4_/CCl_4_ (F), the dashed lines are Nonlinear regression fit curves. (G) Frequency histogram of intensity ratio distribution between groups (CCl_4_/Oil), the dashed lines are Nonlinear regression fit curves. (H) Biological process of Gene Ontology (GO) enrichment analysis of differentially expressed proteins in Figure 2G.


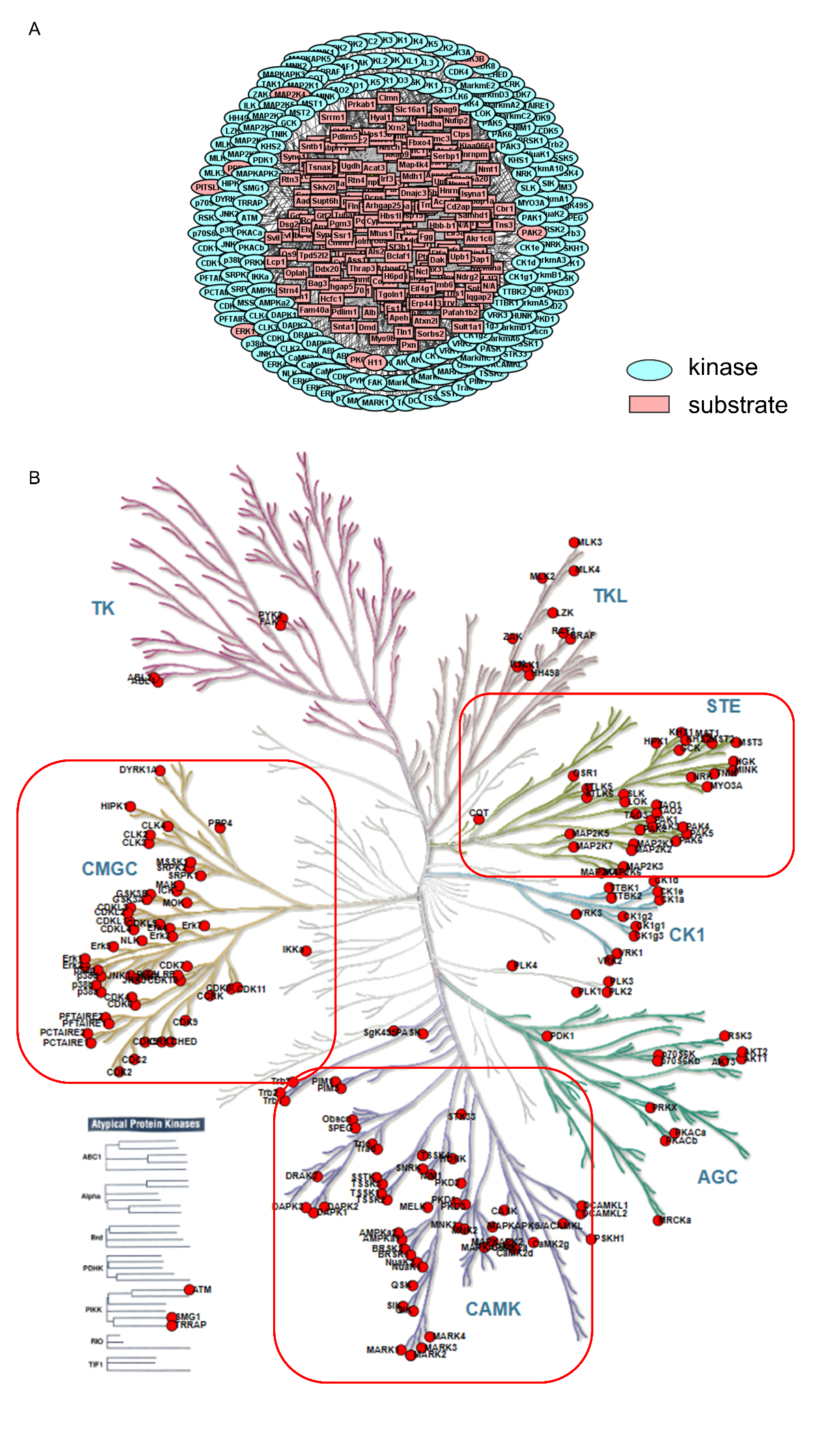


Figure S4. Kinase-substrate predictive analysis. (A) A network map of kinase-substrate predictive analysis of the altered phosphorylation sites in group II in Figure 4A using iGPS software. (B) The kinase tree of the predicted kinase in panel A was drawn. The red boxes represent the families of 13 kinases that were screened in our dataset.


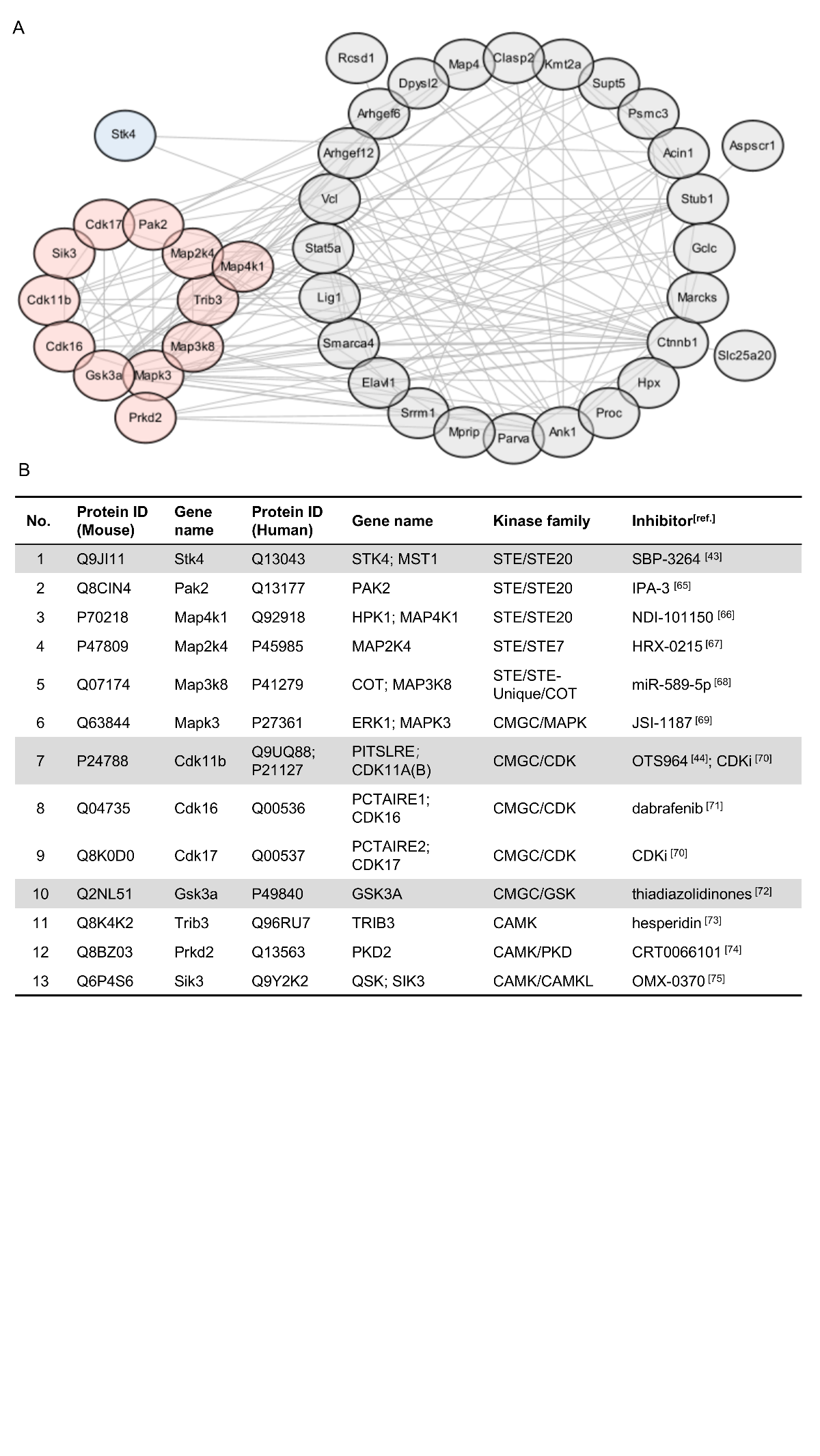


Figure S5. Interaction network analysis of upregulated kinases and their inhibitors. (A) Diagram of the interaction network between kinases and substrates. Blue and red indicate kinases, and gray is the substrate. (B) List of the type of kinase family of the up-regulated kinases and their reported inhibitors.


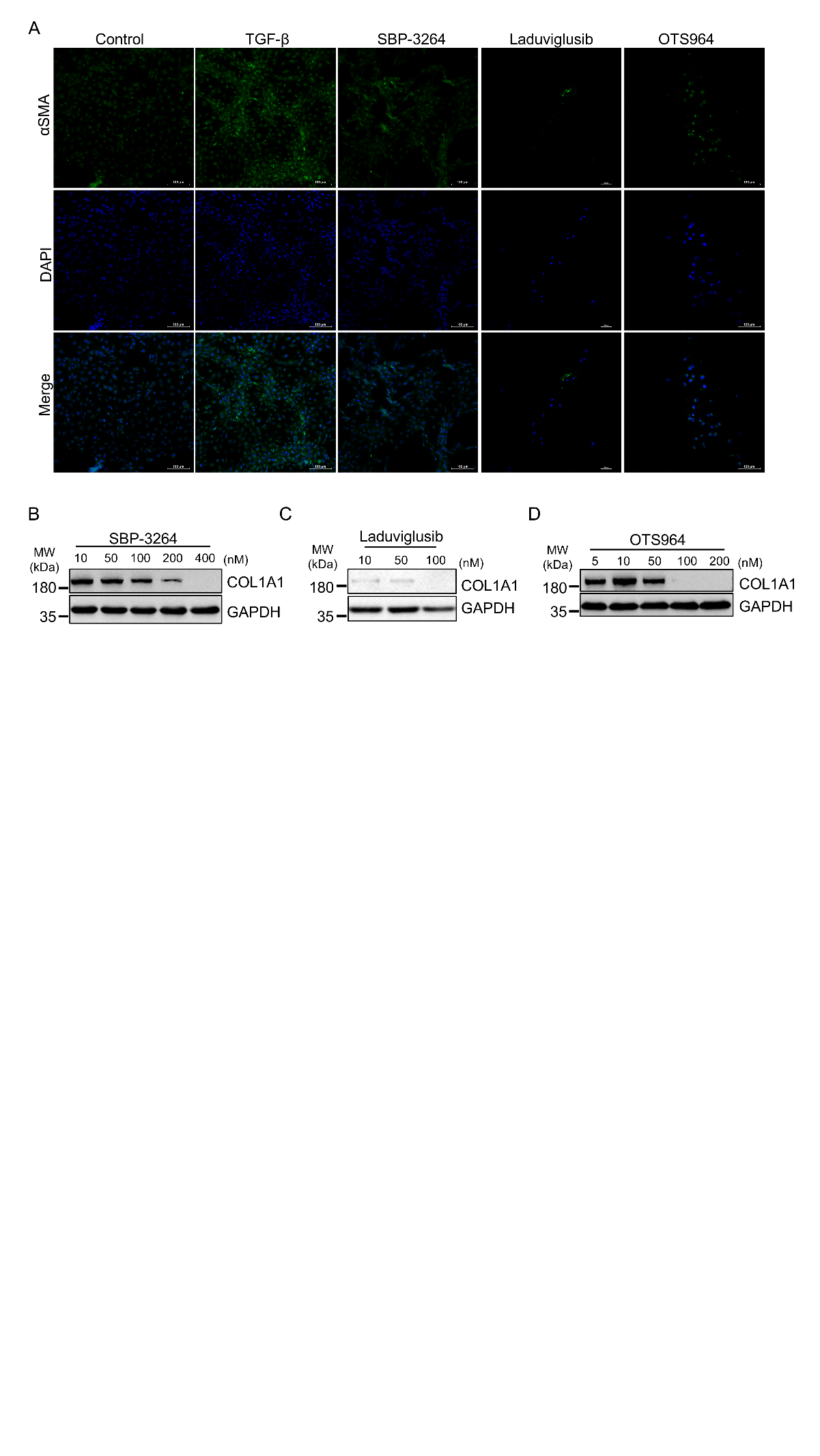


Figure S6. Activation of hepatic stellate cells could be inhibited with kinase inhibitors (A) Immunofluorescence showed that TGF-β stimulation could significantly inhibit the activation of hepatic stellate cells by adding kinase inhibitors, and the production of αSMA was reduced. (B) – (D) The effects of different concentrations of SBP3264 (A), Laduviglusib (B) and OTS964 (C) on collagen Ⅰ production were tested. As the dose of the drug increased, the collagen Ⅰ content decreased.


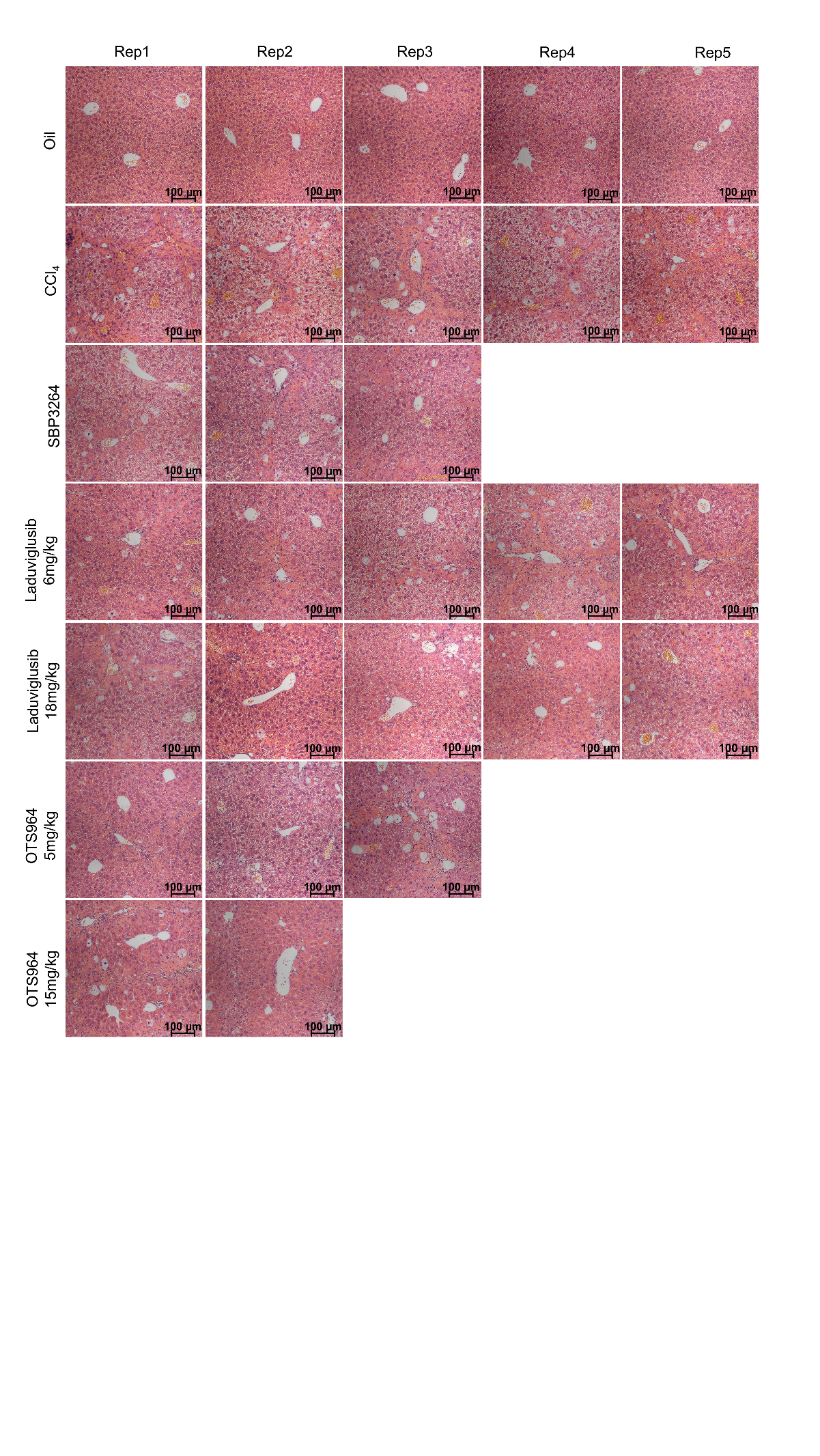


Figure S7. Liver tissues from vehicle- or inhibitors-treated fibrotic mice were stained with HE. There were 5 mice in each group. Due to drug toxicity or drug injection operation, SBP3264 and OTS964 groups had different degrees of death, and the missing part represented the dead mice. The scale is 100 μm.


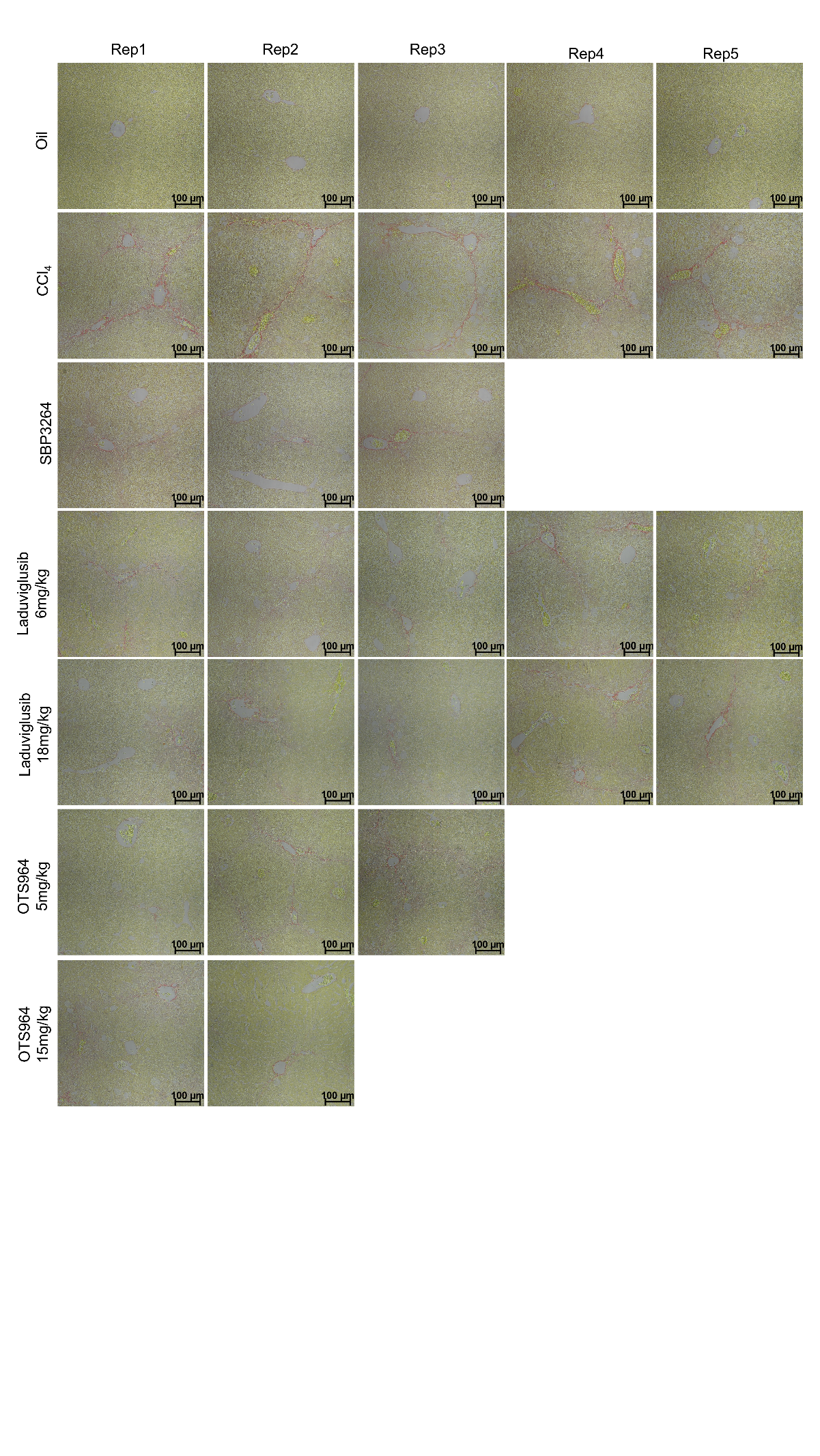


Figure S8. Liver tissues from vehicle- or inhibitors-treated fibrotic mice were stained with Sirius Red. There were 5 mice in each group. Due to drug toxicity or drug injection operation, SBP3264 and OTS964 groups had different degrees of death, and the missing part represented the dead mice. The scale is 100 μm.


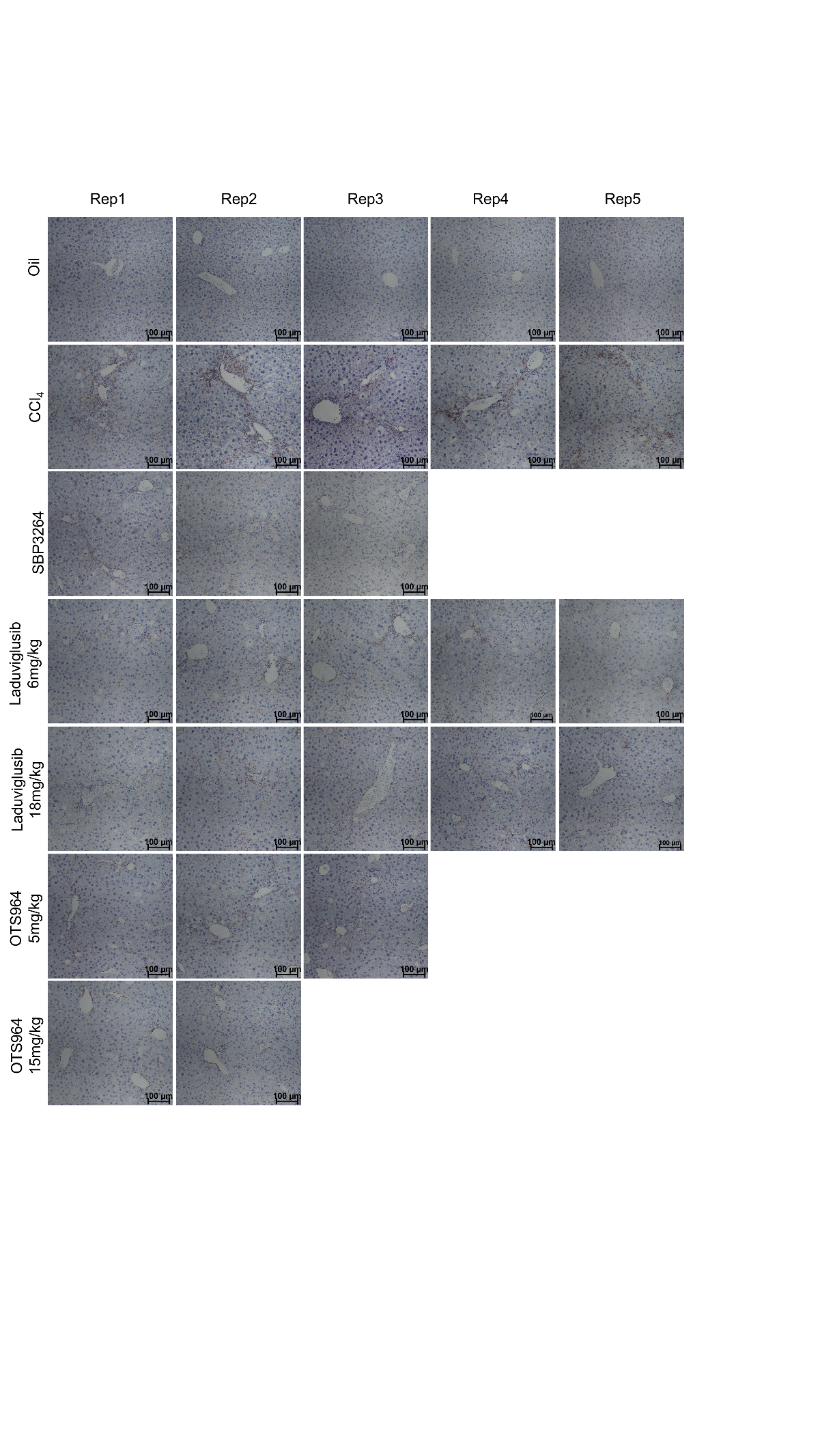


Figure S9. Liver tissues from vehicle- or inhibitors-treated fibrotic mice were stained with αSMA. There were 5 mice in each group. Due to drug toxicity or drug injection operation, SBP3264 and OTS964 groups had different degrees of death, and the missing part represented the dead mice. The scale is 100 μm.


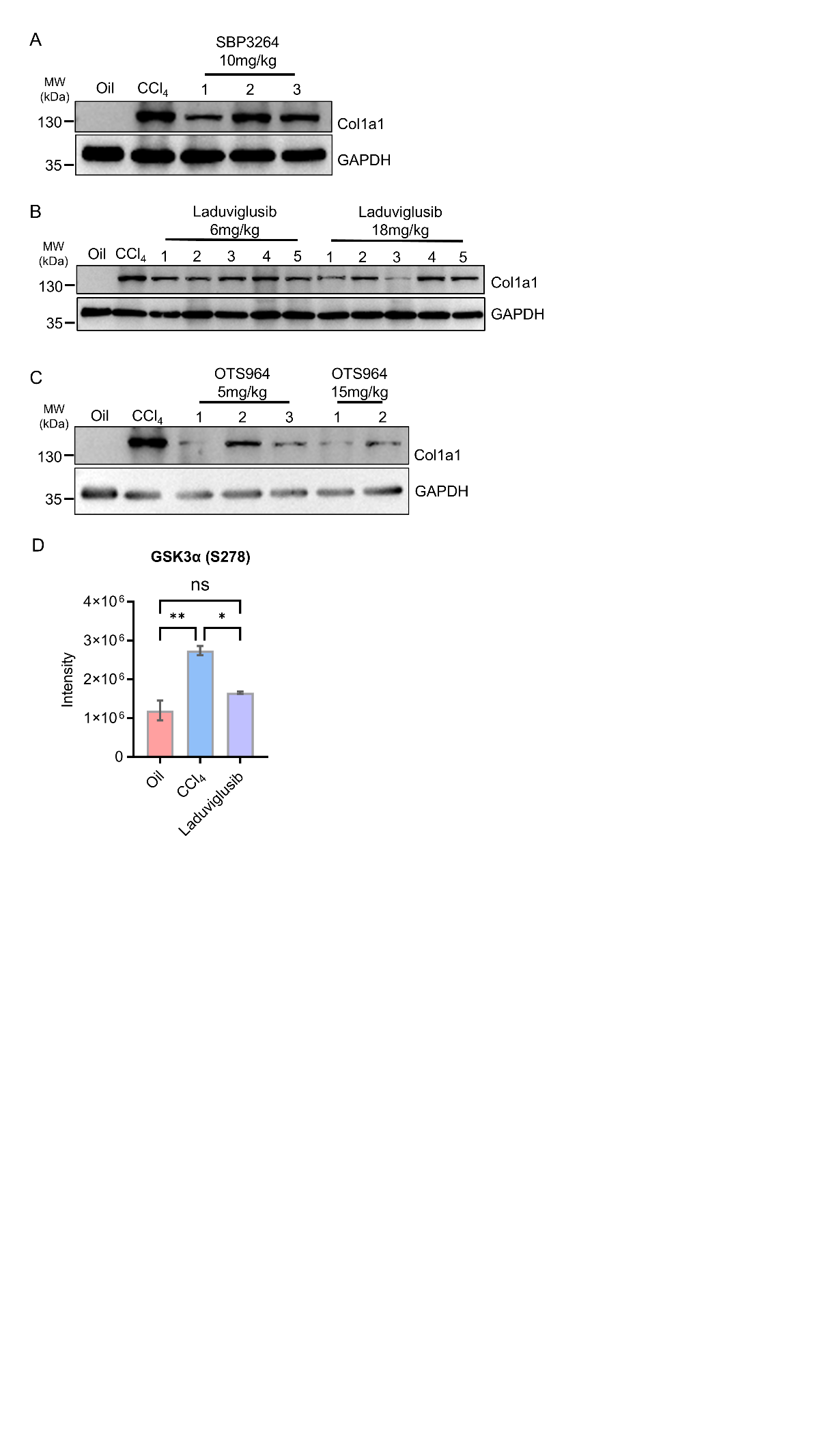


Figure S10. Kinase inhibitors could alleviate the key fibrosis indicator and phosphorylation of target kinase in the mice liver fibrosis. (A) Western blotting assay of collagen I expression in CCl_4_-induced hepatic fibrosis mice after treatment with STK4 inhibitor SBP3264. (B) Western blotting assay of collagen I expression in CCl_4_-induced hepatic fibrosis mice after treatment with GSK3α inhibitor Laduviglusib. (C) Western blotting assay of collagen I expression in CCl_4_-induced hepatic fibrosis mice after treatment with CDK11B inhibitor OTS964. (D) The phosphorylated peptide, “RGTS(p)PRPPEGGLGYSQLGDDDLK”, derived from CDK11B was selected for target detection and quantification, * *p*-value < 0.05, ** *p*-value < 0.01, ns not significant.
